# Detecting axonal injury in individual patients after traumatic brain injury

**DOI:** 10.1093/brain/awaa372

**Published:** 2020-11-30

**Authors:** Amy E Jolly, Maria Bălăeţ, Adriana Azor, Daniel Friedland, Stefano Sandrone, Neil S N Graham, Karl Zimmerman, David J Sharp

**Affiliations:** 1 Clinical, cognitive and computational neuroimaging laboratory (C3NL), Department of Brain Sciences, Faculty of Medicine, Imperial College London, London, W12 0NN, UK; 2 UK Dementia Research Institute Care Research and Technology Centre, Imperial College London and the University of Surrey, London, W12 0NN UK

**Keywords:** traumatic brain injury, diffusion tensor imaging, diffuse axonal injury, diagnostic pipeline

## Abstract

Poor outcomes after traumatic brain injury (TBI) are common yet remain difficult to predict. Diffuse axonal injury is important for outcomes, but its assessment remains limited in the clinical setting. Currently, axonal injury is diagnosed based on clinical presentation, visible damage to the white matter or via surrogate markers of axonal injury such as microbleeds. These do not accurately quantify axonal injury leading to misdiagnosis in a proportion of patients. Diffusion tensor imaging provides a quantitative measure of axonal injury *in vivo*, with fractional anisotropy often used as a proxy for white matter damage. Diffusion imaging has been widely used in TBI but is not routinely applied clinically. This is in part because robust analysis methods to diagnose axonal injury at the individual level have not yet been developed. Here, we present a pipeline for diffusion imaging analysis designed to accurately assess the presence of axonal injury in large white matter tracts in individuals. Average fractional anisotropy is calculated from tracts selected on the basis of high test-retest reliability, good anatomical coverage and their association to cognitive and clinical impairments after TBI. We test our pipeline for common methodological issues such as the impact of varying control sample sizes, focal lesions and age-related changes to demonstrate high specificity, sensitivity and test-retest reliability. We assess 92 patients with moderate-severe TBI in the chronic phase (≥6 months post-injury), 25 patients in the subacute phase (10 days to 6 weeks post-injury) with 6-month follow-up and a large control cohort (*n = *103). Evidence of axonal injury is identified in 52% of chronic and 28% of subacute patients. Those classified with axonal injury had significantly poorer cognitive and functional outcomes than those without, a difference not seen for focal lesions or microbleeds. Almost a third of patients with unremarkable standard MRIs had evidence of axonal injury, whilst 40% of patients with visible microbleeds had no diffusion evidence of axonal injury. More diffusion abnormality was seen with greater time since injury, across individuals at various chronic injury times and within individuals between subacute and 6-month scans. We provide evidence that this pipeline can be used to diagnose axonal injury in individual patients at subacute and chronic time points, and that diffusion MRI provides a sensitive and complementary measure when compared to susceptibility weighted imaging, which measures diffuse vascular injury. Guidelines for the implementation of this pipeline in a clinical setting are discussed.

## Teaser

Jolly *et al.* develop a diffusion MRI pipeline for the diagnosis of axonal injury in individual patients with traumatic brain injury. They demonstrate the sensitivity of this pipeline for detecting axonal injury, show its relationship to clinical measures, and provide practical guidelines for its use in subacute and chronic TBI.

## Introduction

Long-term outcomes are often poor after traumatic brain injury (TBI) and it is challenging to understand the causes of its varying effects (Colantonio *et al.*, 2004; Menon and Zahed, 2009; McMillan *et al.*, 2011). A key reason for this is that diffuse axonal injury (DAI) is difficult to measure clinically. This is important because DAI plays a fundamental role in the pathophysiology of TBI and is an important predictor of outcome (Sidaros *et al.*, 2008). Axons and axoplasmic membranes are injured by initial shearing forces produced at the time of injury (Smith *et al.*, 2003). This impairs axonal transport, disrupts brain network function and results in delayed axonal damage. At present, we have limited diagnostic tools to measure axonal injury. A key goal for TBI research is to develop and validate sensitive and specific measures of DAI that can robustly identify the extent of injury.

Currently, the diagnosis of diffuse axonal injury after TBI relies upon the clinical presentation of a patient and the presence of observable damage to the white matter using CT or MRI imaging (Adams *et al.*, 1989; Marshall *et al.*, 1991; Gallagher *et al.*, 2007). For example, the Adams diffuse axonal injury classification provides useful guidelines for determining the presence and severity of DAI (Adams *et al.*, 1989). However, this method is likely to underestimate DAI when conventional imaging approaches are used (Cicuendez *et al.*, 2017) as they do not provide a sensitive measure of the microstructural changes to white matter that are present, but not visible on routine MRI (Sharp *et al.*, 2014; Hellstrøm *et al.*, 2017).

Advances in MRI now allow axonal injury to be sensitively detected after TBI (Mac Donald *et al.*, 2007*a*, *b*, 2011; Kinnunen *et al.*, 2011; Bonnelle *et al.*, 2012). The development of diffusion tensor imaging (DTI) has provided a quantitative method to assess white matter structure *in vivo* with measures of fractional anisotropy (FA) used as a proxy for white matter integrity (Mac Donald *et al*., 2007*a*). This technique has demonstrated an improved ability to detect and quantify diffuse axonal injury in patients (Basser and Pierpaoli 1996; Sidaros *et al.*, 2008) as demonstrated by the identification of abnormalities in the context of an otherwise normal scan (Corbo and Tripathi, 2004; Hashim *et al.*, 2017; Hellstrøm *et al.*, 2017). Furthermore, DTI has been shown to be a valid measure of white matter integrity after TBI in animal models of traumatic injury (Mac Donald *et al.*, 2007*a*)and provides a sensitive structural measure of damage to white matter that becomes abnormal in the first hours after injury (Mac Donald *et al*., 2007*b*) and remains abnormal into the chronic phase (Laitinen *et al.*, 2015). Importantly, DTI has helped to predict long-term clinical outcomes after TBI (Sidaros *et al.*, 2008; Kumar *et al.*, 2009) and can be used to explain abnormalities of brain function seen after injury (Sharp *et al.*, 2014). The location of diffusion abnormalities has been shown to predict many types of cognitive problems that patients often experience after TBI, particularly in large long-range white matter connections that are integral in supporting functional networks of cognition (Hellyer *et al.*, 2013; Sharp *et al.*, 2014; Fagerholm *et al.*, 2015).

Despite the clear evidence of the utility of diffusion MRI in the assessment of TBI, it is not widely used clinically. A key challenge is to define analysis methods that are robust to the challenges of clinical imaging data and that can be used to provide diagnostic information at the level of individual patients. Here we present a pipeline for diffusion imaging that is robust to common methodological challenges, including the impact of focal lesions, variation in FA with age and test-retest reliability issues. The pipeline produces results with high sensitivity and specificity, test-retest reliability and that correlate with common clinical outcomes after TBI. This is achieved using measures of fractional anisotropy (FA) in large white matter tracts to calculate whether significantly abnormal FA is observed in individual TBI patients compared to healthy individuals.

We first apply a data-driven approach to identify large white matter tracts with high reliability across time by investigating test-retest reliability of FA at three time points across a 2-year period. We select tracts that include association, projection and commissural pathways to provide good anatomical coverage. We demonstrate the rates of axonal injury identified in individual patients and investigate associations with cognitive, psychiatric and functional outcomes. Finally, we examine the impact of age, sample size, time since injury and focal lesions on our diagnostic results to provide pragmatic guidelines for the use of this pipeline in subacute and chronic TBI.

## Materials and methods

### Participants

Data from 92 patients [mean age 42.9, 12.02 standard deviation (SD) years, 19 females] and 103 healthy controls (mean age 44.5, 14.76 SD years, 35 female) were included in this study. Patients and controls had no history of psychiatric illness, drug or alcohol dependence and were not undergoing any litigation. Controls had no neurological history including traumatic brain injury. There was no significant difference in age between the two groups [*t*(192) = 0.84, *P = *0.4]. All patients were classified as moderate-severe as defined by the Mayo criteria (Malec *et al.*, 2007) and were recruited from Neurology outpatient clinics at St Mary’s hospital, London, within the chronic phase of their injury (mean time since injury 130 months, range 6–497 months). Further information regarding patients' mechanism of injury and current medications can be found in Supplementary Table 1. All patients and controls underwent advanced structural MRI. Of the 92 patients included, 16 had no visible evidence of injury on routine MRI (e.g. contusion or microbleeds), 11 had evidence of microbleeds, 36 had a focal contusion and 29 patients had both contusions and microbleeds as classified by a consultant neuro-radiologist. All patients completed standard neuropsychological testing and questionnaires to determine functional outcomes after injury. A subgroup of the controls (*n = *35) also completed neuropsychological testing. Written informed consent was obtained for all participants in accordance with the Declaration of Helsinki. The studies were approved by the West London and GTAC Research Ethics Committee (14/LO/0067, 13/LO/1678, 14/LO/1998).

To investigate the impact of time since injury, an additional 25 patients with moderate-severe TBI (mean age 49.36, 13.45 SD years, seven females) with subacute longitudinal diffusion weighted imaging data collected on the same scanner were included. Patients within this subset were scanned at subacute (10 days to 6 weeks) and chronic (≥6 months) time points. Patients were scanned no less than 10 days post-injury to allow for stabilization of FA measures, which can be transiently elevated soon after injury (Mac Donald *et al*., 2007*b*). Further information regarding time since injury, scanning interval and mechanism can be found in Supplementary Table 2.

### Neuropsychological and functional outcomes assessment

A standardized neuropsychological battery previously used to investigate cognitive impairment after TBI was implemented (Kinnunen *et al.*, 2011; De Simoni *et al.*, 2018). Tests were designed to investigate executive function, memory, processing speed and reasoning and included; the Trail Making Test (TMT) and Delis-Kaplan Executive Function Systems (D-KEFS) Colour-Word Interference (Stroop) test to assess processing speed and executive function (Delis *et al.*, 2001); the Peoples Test (PT) from the Doors and Peoples Test to measure episodic memory (Weschler, 1945) as well as the Weschler Abbreviated Scale for Intelligence (WASI) Matrix reasoning task to assess abstract reasoning ability (Weschler, 1945). Self-report measures of functional outcomes, mood and sleep after injury were assessed using the Glasgow Outcome Scale-Extended (GOSE) (Jennett and Bond, 1975; Jennett *et al.*, 1981), the Hospital Anxiety And Depression Scale (HADS) (Snaith, 2003) and the Pittsburgh Quality of Sleep Index (PSQI) (Buysse *et al.*, 1989).

### Structural MRI acquisition

Structural MRI was acquired using a 3 T Siemens Magnetom Verio Syngo with a 32-channel head coil. All patients and controls were scanned using the same MRI machine and acquisition parameters. Structural MRI included a high-resolution T_1_-weighted MPRAGE (106 1-mm thick transverse slices, repetition time = 2300 ms, echo time = 2.98 ms, flip angle = 9°, in-plane resolution *=* 1 × 1 mm, matrix size = 256 × 256, field of view = 25.6 × 25.6 cm), diffusion weighted imaging (64 directions, b = 100 s/mm^2^ with four interleaved b = 0 s/mm^2^, echo time/repetition time = 103/9, 500 ms, 64 contiguous slices, field of view 256 mm, voxel size 2 mm^3^) and FLAIR to identify focal lesions.

### Delineation of focal lesion location

In-house software (ImSeg) was used to delineate focal contusions and areas of missing brain as lesion masks on T_1_-weighted MPRAGE images. This allows for the identification and subsequent exclusion of areas in a white matter tract that may bias the overall mean FA that are not representative of axonal injury. Lesion masks were manually drawn with FLAIR images overlaid to aid identification of boundaries of lesioned areas. All masks were drawn in native space and subsequently registered to standard space. This was achieved by first registering an individual’s T_1_ to MNI152 1 mm space using FIMRIB’s linear image registration tool (FLIRT) (Jenkinson and Smith, 2001; Jenkinson *et al.*, 2002) and applying the resulting transformation matrix to lesion masks to warp into standard space. Masks were subsequently thresholded and rebinarized.

### Statistical analysis

#### Diffusion tensor imaging group-level analysis

Diffusion-weighted images were processed using FSL’s diffusion toolkit (FDT) and standard preprocessing techniques (Behrens *et al.*, 2003). Specifically, eddy current correction was applied to diffusion-weighted images to correct for eddy current distortions and movement during acquisition. BVECs were then rotated using the eddy correction output and brain extraction (BET) was then applied to strip the skull from the eddy corrected data. Tensor fitting was achieved using DTIFIT with a weighted least squares approach to derive FA maps. Individual FA maps constructed using DTIFIT were then registered to standard MNI152 space using the FIMRIB 1 mm FA atlas in tract-based spatial statistics (TBSS) (Smith *et al*., 2006). All FA maps concatenated and in standard space were subsequently skeletonized at 0.2 threshold. This ensures only FA from the centre of the white matter tracts remain in order to avoid partial volume effects. To extract mean FA values for each tract region of interest for each patient and control, the concatenated skeletonized images were separated using fslsplit into their individualized FA skeleton maps. Tract region of interest masks were then intersected with each individual skeletonized FA map to extract the mean FA for each individual within the selected regions of interest using fslstats. As a summary measure to capture FA across the whole white mater, whole brain mean FA was also extracted for each individual by intersecting skeletonized FA maps with the whole brain mean FA skeleton mask derived from TBSS. This provides further investigation of the presence of generalized reductions in FA that may not be detected by the individual regions of interest selected but remains an important factor in relation to cognitive and clinical outcomes (Kinnunen *et al.*, 2011; Jenkins *et al.*, 2018).

A group-level comparison of FA for patients and controls was applied using a repeated measures ANOVA across tract regions of interest (excluding whole brain FA) to determine whether the patient cohort demonstrated evidence of axonal injury. To investigate the presence of axonal injury at the individual patient level, FA for all controls and the patient of interest were used in the DTI diagnostic pipeline. A summary of the regions of interest selected for use in the pipeline and the methodology to detect axonal injury are described in the following sections.

#### Pipeline for the diagnosis of axonal injury in individual patients

**Figure 1:**
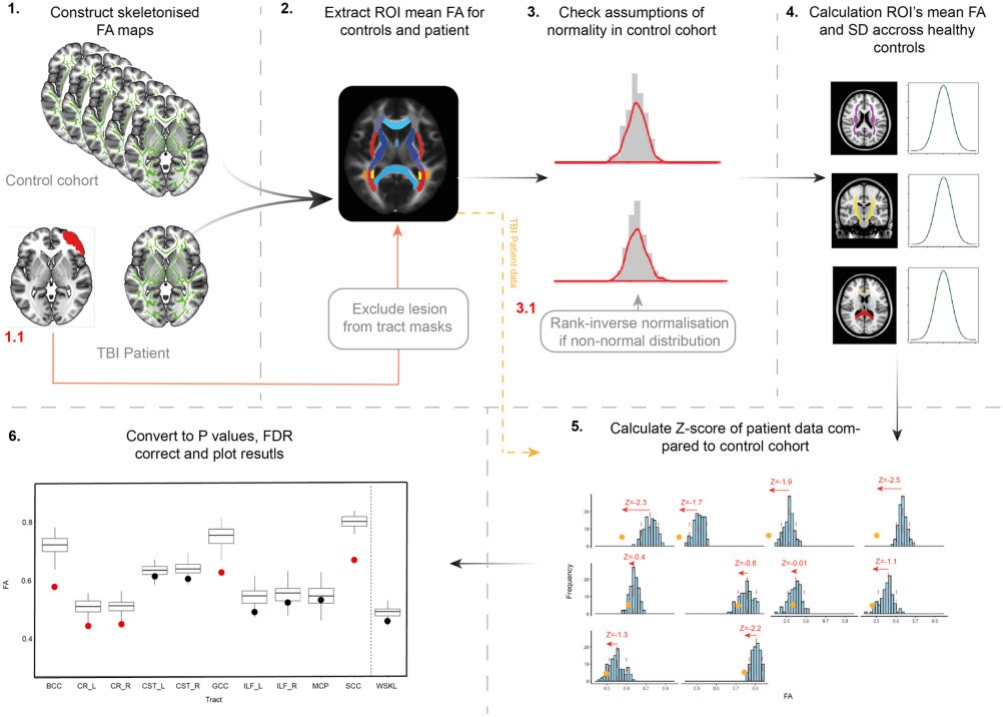
**DTI diagnostic pipeline processing steps.** 1: Skeletonized FA maps are derived for the patient of interest and the comparative control group using TBSS. 1.1: If a patient has evidence of contusion or missing brain, these areas are manually delineated using in house software (ImSeg) to produce a lesion mask. The lesion mask is then registered to standard MNI152 1 mm space and subtracted from tract masks so that these regions are excluded from influencing subsequent FA measures. 2: Skeletonized FA maps from controls and individual patients are tract masks to extract mean FA value for each region of interest (ROI) for each individual. 3: Control FA distributions are assessed for assumptions of normality using histograms, QQ plots and Shapiro-Wilks tests. 3.1: If assumptions of normality are violated, rank-inverse normalization is applied. 4: Control group mean FA and SD for each region of interest is then calculated for use in *Z*-scoring. 5: *Z*-scores for each patient are calculated. 6: *Z*-scores for a patient are then converted to *P*-values (95% CI, two tailed) and corrected for multiple comparisons using FDR. Whole brain FA skeleton is calculated as a stand-alone test. Individual patient data are then plotted against control group and significantly lower FA in patients are highlighted in red for ease of interpretation.

A summary of the methods used in the axonal injury DTI pipeline can be found in Fig. 1. In summary:

- Skeletonized FA maps of the healthy control cohort and individual TBI patient are constructed using the TBSS pipeline. If there is evidence of contusion/missing brain in the TBI patient, a lesion mask is manually delineated using ImSeg. The binarized lesion mask is then registered to standard space by first registering the subject’s T_1_ to MNI152 1 mm space using FLIRT and applying the transformation matrix to the lesion mask. The standardized lesion mask is then used to exclude lesioned areas from the tract regions of interest by subtracting from all tract region of interest masks using fslmaths. In this way, any tract region of interest where an area of lesion overlaps is redefined to exclude this area from FA sampling. The mean FA for the resulting tract region of interest masks is extracted for both the patient and the control cohort to allow for comparison of FA and used in the subsequent steps of the tool. If the mask includes a correction for the presence of a lesion, the control group FA is specifically calculated for this corrected mask.
- Skeletonized FA maps (of the patient and control group) are intersected with tract region of interest masks to extract mean FA for each region of interest for each participant.
- Checks for assumptions of normality are performed across each tract to determine whether parametric methods are appropriate. Distribution of FA and QQ plots in the healthy population are plotted and Shapiro-Wilk tests applied. If assumptions of normality are not met, a rank-inverse normalization can be applied to the data (applying the transformation to the control group and patient together) to provide ranked data with normal distribution.
- Next, the mean and standard deviation of FA for each tract region of interest across the control cohort is then calculated.
- Patient *Z*-scores are then calculated for each region of interest by subtracting the control groups FA from the individual patients and dividing by the control groups standard deviation [*z =* (*x −* $\bar{x})$/*ρ*]. A negative *Z*-score indicates a patients FA is lower than the control means.
- *Z*-scores are converted to *P*-values [two-tailed, with 95% confidence interval (CI)] and corrected for multiple comparisons across all tract regions of interest investigated using false discovery rate (FDR). Whole brain FA skeleton values extracted using the whole brain mean FA skeleton mask are calculated as a stand-alone summary measure and is therefore not included in multiple comparisons correction. The patient FA is then plotted against the control cohort for each tract region of interest to demonstrate abnormal tracts (e.g. tracts that have significantly lower FA in the patient compared to the control group, *P <* 0.05, FDR corrected) for ease of interpretation for the user. Code and example data are available for use (https://github.com/ImperialCollegeLondon/c3nl-home).

#### Selection of white matter tract regions of interest for use in diagnostic pipeline

Forty-six white matter tract regions of interest were considered for use in the diagnostic pipeline (Supplementary Table 3). Tracts were derived from the ICBM-81-DTI white matter atlas and included cortical and subcortical white matter projections. Details regarding the delineation of these regions have previously been reported (Oishi *et al.*, 2008). To select the most reliable tracts with good sensitivity to axonal injury and high clinical relevance to impairments after TBI, a set of criteria were applied in a stepwise data-driven approach. These consisted of: (i) the exclusion of small tracts (tracts with number of voxels below median of all candidate tracts); (ii) the identification of high reliability as quantified using intra-class correlation coefficient (ICC); (iii) good anatomical coverage and sampling of association, projection and commissural white matter projections (Mori *et al.*, 2008); and (iv) clinical relevance to traumatic brain injury as defined by prior evidence of relationship to motor, cognitive and functional outcomes. We aimed to select ∼10 tracts, to provide balance between good white matter coverage and power to detect axonal injury after correction for multiple comparisons. To allow comparison across hemispheres, tracts included both left and right hemispheres where possible.

First, median tract size (number of voxels) was calculated across all 46 regions of interest. Tracts with a total number of voxels lower than the median were excluded from the candidate pool. This ensures only the largest tracts are considered as previous evidence indicates erroneous and variable tensor fitting (thus low reliability) specific to FA in smaller tracts (Sairanen *et al.*, 2017). The remaining candidate tracts were then assessed for test-retest reliability by examining the average ICC of FA in 20 healthy controls scanned across three time points (Years 1, 2 and 3). An ICC value above 0.6 indicates good reliability (Cicchetti, 1994), we therefore selected this as our threshold for the inclusion or exclusion of tracts. Next, the remaining tracts were examined to determine good coverage of association, commissural and projection pathways as well as their relevance to TBI. Providing good anatomical coverage of the white matter in all directions ensures we can detect axonal injury due to rotational/shearing forces applied to the brain within a given direction (Ghajari *et al.*, 2017). Additionally, white matter tracts relating to motor and cognitive impairments as well as functional outcomes after TBI when damaged (Kinnunen *et al.*, 2011; De Simoni *et al.*, 2018; Jenkins *et al.*, 2018) were considered as priority to retain if more than 20% of candidate tracts remained after application of previous criteria.

#### Testing the effect of age on axonal injury diagnosis

Normal ageing is associated with changes in FA (Pfefferbaum *et al.*, 2000) and may therefore impact upon diagnosis of normal versus abnormal (injured) tracts if the patient and control cohort differs greatly in age. A repeated measures ANOVA was therefore applied to control data to examine if there was a main effect of age on FA. A one-way ANOVA was also applied to whole brain FA to examine the impact of age. We quantified the impact of ageing on our axonal injury diagnosis by comparing: (i) diagnosis without accounting for age using all controls; and (ii) diagnosis using control data with the effect of age regressed out of the control and patient data. In the first method, each of the 92 patients were individually compared to all healthy controls using the pipeline. The number of patients diagnosed as having abnormal white matter (as defined by one or more significantly abnormal tracts, *P < *0.05 FDR corrected, excluding the whole brain measure) were quantified. In the second method, all 92 patients and 103 controls were included in a multiple linear regression to regress age and age^2^ out of FA allowing us to account for any non-linear relationships between age and FA. The residuals from this regression were then used and each individual patient was compared to all 103 controls using the diagnostic pipeline.

#### Testing the impact of control sample size on diagnosis

An important issue is the sample size of controls required to produce stable diagnostic performance. As the diagnosis of evidence of axonal injury is derived using *Z*-scores, potential bias arises from small control sample sizes. We therefore investigated the effect of varying sample size on diagnostic rates. We randomly subsampled the control cohort to produce samples of 10, 20, 30, 40 and 50. Mean FA and its standard deviation from each region of interest was then used to reassess each of the 92 patients. This process was repeated 100 times for each control sample size and each individual patient to improve reliability of the estimate. The reliability of diagnosis within a given sample size was calculated using ICC of *P*-values (FDR corrected) derived using the pipeline across all of the 100 sample size iterations for each patient. A high ICC indicates that the results derived for patients across all 100 subsamples of a control *n* are reliable.

We then calculated rates of type 1 and type 2 errors at varying sample sizes by comparing diagnostic results to those derived using the whole control cohort. Taking the results of all 100 iterations for a patient, we deemed an abnormal tract to be present if more than 5% of the 100 test iterations identified it as such. In this way, we assumed a tract to be deemed abnormal if diagnosed as such above an expected 5% false positive rate when testing at a given sample size 100 times. We quantified the number of false positives (a patient is identified as having evidence of a white matter abnormality in at least one tract in the smaller sample size but not with the whole control cohort), false negatives (a patient is not deemed abnormal in contrast to the whole control cohort classification), true negatives (a patient is not deemed abnormal in agreement with the whole control cohort classification) and true positives (a patient is deemed abnormal in agreement with the whole control cohort classification). Finally we calculated measures of sensitivity (accurately identifying a patient as abnormal in the context of their diagnosis using the whole control cohort) and specificity (accurately identifying a patient as normal in the context of their diagnosis using the whole control cohort), positive predictive values (PPV) (the probability of patients being identified as abnormal using the smaller sample size are also diagnosed as abnormal using all controls) and negative predictive values as measures of ‘diagnostic accuracy’ in the context of a smaller sample size compared to diagnosis derived from the whole control cohort.

#### Testing the specificity of the diagnostic pipeline

To test the specificity of the diagnostic pipeline, we then applied the diagnostic process to each of the 103 healthy controls using a leave-one-out approach. This allowed the rate of false positive tracts to be identified. Diagnosis was performed on non-age regressed data. If the tool had low specificity to axonal injury in TBI we would expect to see false positive rates exceed an acceptable rate of 5% in healthy controls.

#### Neuropsychological assessment and functional outcomes

Performance on individual neuropsychological tests were compared between TBI patients and controls. As data were not normally distributed, non-parametric tests (Wilcoxon rank sum tests) were used. To account for the issue of multiple comparisons an FDR correction was applied within each cognitive domain assessed. Composite scores for each cognitive domain (executive function, information processing, reasoning and episodic memory) were calculated by averaging across tasks within a given cognitive domain. Scores were then normalized for comparison across domains. Patients diagnosed with a DTI abnormality as determined using the diagnostic pipeline were compared to those with no abnormality and healthy controls using one-way ANOVAs. Functional outcomes, mood and sleep assessed using the GOSE, HADS and PSQI, respectively, were also compared between patients with or without abnormalities. In addition, to assess whether certain tracts had a greater association to cognitive composite measures, correlational analysis was performed between each tract and each composite measure for patients. Multiple linear regression was not applied because of high collinearity between tracts. Results from correlational analysis were therefore corrected for multiple comparisons using an FDR approach. Finally, to determine whether any differences in neuropsychological and functional outcomes were specific to diagnosis of axonal injury using our pipeline, scores across cognitive domains and functional outcomes were also compared between patients based on visible damage on MRI. Therefore, cognitive scores and GOSE measures were compared between patients with and without microbleeds or patients with and without contusions (as defined by the consultant neuroradiologist) using unpaired two-tailed *t*-tests with FDR correction.

#### Testing the effect of time since injury on diagnosis

An important issue when examining FA alterations after TBI is the effect of time since injury. After the initial injury, dynamic changes to FA can occur leading to elevated FA acutely and progressive reductions chronically (Werner and Engelhard, 2007). These longitudinal alterations may impact upon the detection of abnormalities across different phases of injury. From a clinical perspective, detection of axonal injury at an acute time point may be particularly beneficial in guiding the initial management of patients but may be challenging if fluctuations in FA are present and reliability is low. We therefore investigated the relationship between time since injury and diagnosis with our pipeline in two patient cohorts. First, in a between subject analysis, we investigated whether significant differences in time since injury was present in our large cohort of chronic patients (*n = *92) identified with or without abnormalities using an unpaired two-tailed *t*-test. We then applied bivariate correlations to determine whether there was a significant relationship between time since injury and FA across all tracts and mean whole brain skeleton in patients after correcting for age and multiple comparisons using FDR.

Next, we investigated the reliability of the diagnostic pipeline in the subacute phase of injury using an additional cohort of patients (*n = *25) scanned during subacute (10 days to 6 weeks) and chronic phases (∼6 months) of injury. We examined the diagnostic results of patients using the pipeline and investigated the reliability of diagnosis by calculating the ICCs of tract abnormalities between the two time points (as quantified by *Z*-score and *P-*values derived from the diagnostic pipeline and FDR corrected). Next, we compared measures of tract FA between (i) the subacute patient time point and controls; and (ii) the 6-month follow-up (chronic) time point for patients and controls using factorial ANOVA. Comparisons between time points and controls were repeated for whole brain skeleton FA using one-way ANOVA. Finally, comparison within patients between the subacute and chronic time points were made using a repeated measures ANOVA for tract data and *t*-test for whole brain skeleton FA. To ensure comparisons within subjects across the two time points are consistent (Squarcina et al., 2012), we excluded areas of lesion (inclusive of oedema) identified at the subacute phase of injury from the analysis at both time points.

### Data availability

Raw data were generated at Imperial College London. Derived data supporting the findings of this study are available from the corresponding author on request.

## Results

### Tract selection

**Figure 2:**
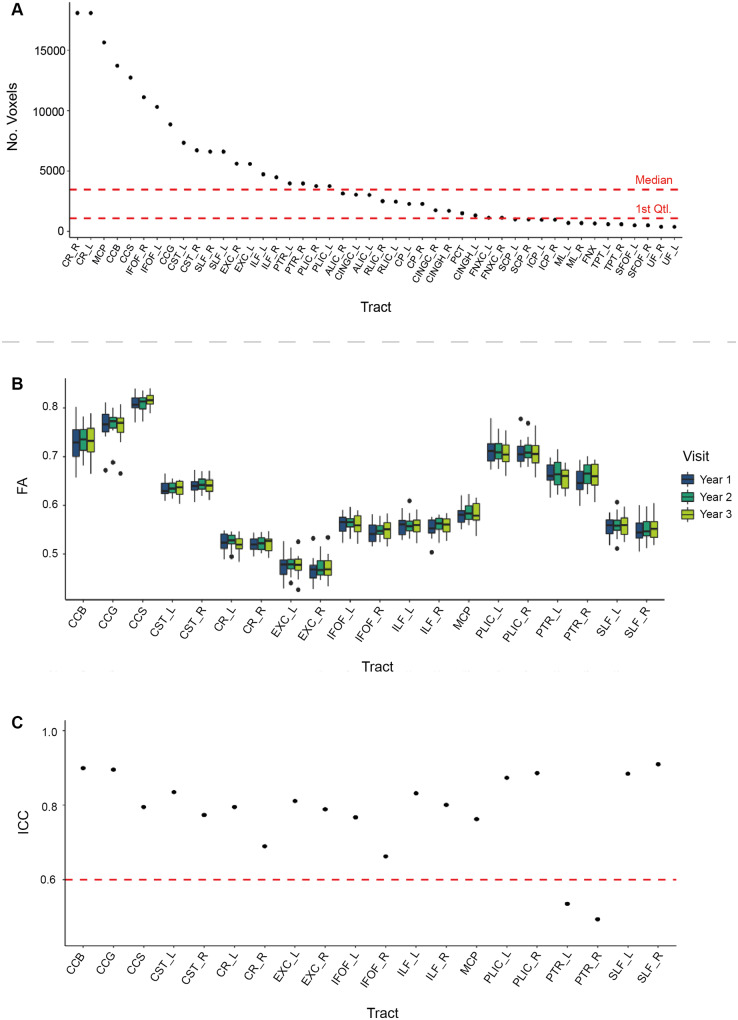
**Data-driven tract selection.** (**A**) Voxel size of all 46 candidate tracts ordered from largest to smallest. Median and first interquartile range demonstrated on red dashed lines. (**B**) Mean FA in each of the remaining candidate tracts across a longitudinal healthy control population (*n =* 20). (**C**) Reliability of tracts calculated using ICC using longitudinal data from healthy control subset. See Supplementary Table 3 for tract abbreviations and associated acronyms.

Of the 46 candidate tract regions of interest considered, seven tracts (10 regions of interest) were selected for use in the diagnostic pipeline after application of a stepwise selection process. First, we considered the size of the tracts. FA calculated from smaller tracts can be unreliable, in part because of errors in tensor construction (Sairanen *et al.*, 2017) and problems skeletonizing the region of interest leading to partial volume effects (Smith *et al.*, 2006; Bach *et al.*, 2014). Hence, we focused analysis on the largest tracts in our set of regions of interest by excluding those smaller than the median voxel size, leaving a total of 20 regions of interest (Fig. 2A).

Next we calculated the reliability of these tracts by measuring the test-retest reliability of FA using data from 20 healthy controls scanned across three time points ∼12 months apart (Fig. 2B). ICCs were calculated to quantify reliability and tracts with a good ICC (>0.6) were retained (Cicchetti, 1994). These criteria led to the posterior thalamic radiations being removed (Fig. 2C). Eleven tracts (18 separate regions of interest) remained after these criteria were applied. FA values within these tracts were normally distributed as defined using the Shapiro-Wilks test of normality (*P > *0.05) confirming that the use of parametric *Z*-scoring to identify abnormalities was appropriate (Supplementary Fig. 1).

**Figure 3:**
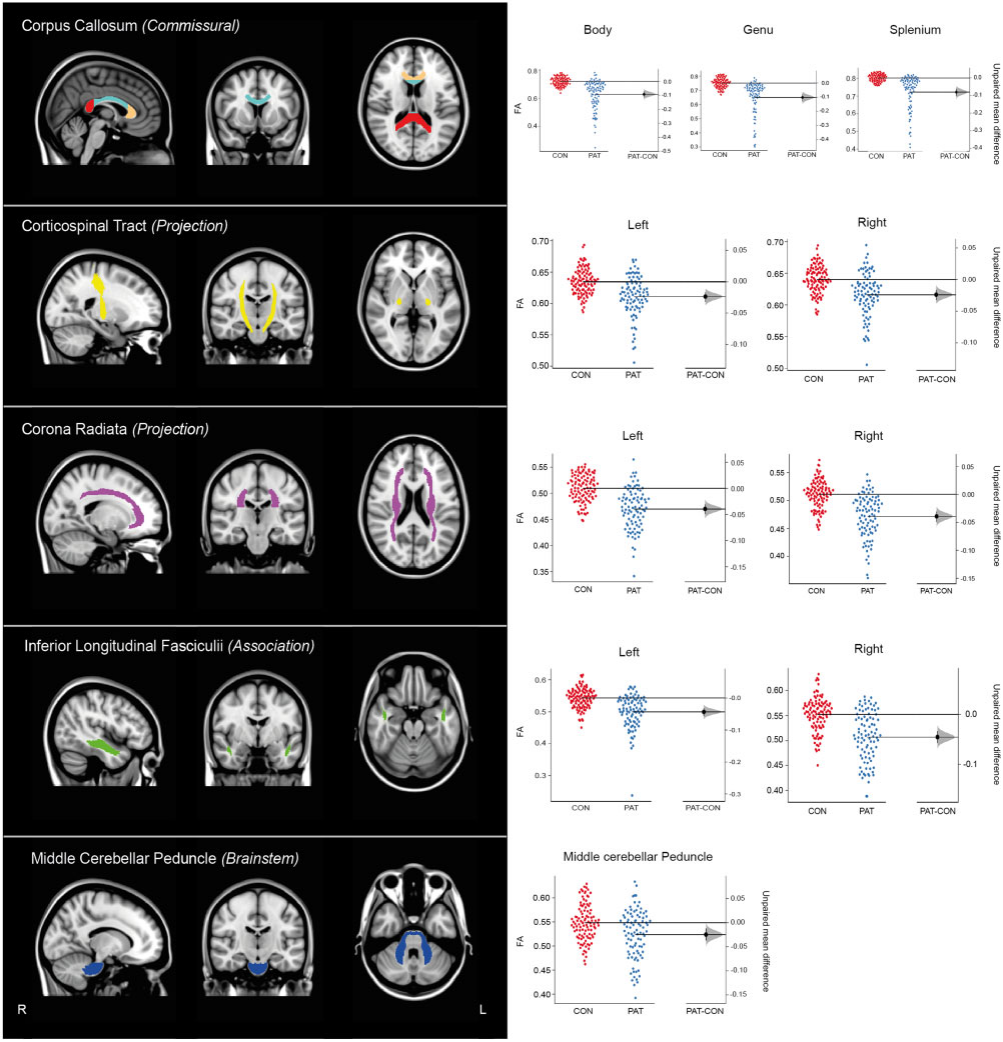
**Tracts selected for use in diagnostic pipeline.** *Left*: Rendering of tracts selected for use in the DTI pipeline. The corpus callosum demonstrates the three subdivisions as: red = splenium, blue = body, and orange = genu. L = left; R = right. *Right*: Estimation plots representing individual mean FA values for TBI patients (PAT) and controls (CON). Black lines represent group level means. A measure of effect size and its 95% CIs calculated through non-parametric bootstrap resampling are plotted on the vertical bar on a separate but aligned axes (Ho *et al*., 2012).

From these 18 regions of interest, 10 were selected to provide sampling of the projection, association and commissural pathways that are commonly affected after TBI and relate to the motor, cognitive and emotional symptoms often observed. Tracts selected were: the body, genu and splenium of the corpus callosum (CCB, CCG, CCS), the corticospinal tract (left and right; CST_L, CST_R), the corona radiata (left and right; CR_L, CR_R), the inferior longitudinal fasciculi (left and right; ILF_L, ILF_R) and the middle cerebellar peduncle (MCP) (Fig. 3A). The corpus callosum is commonly disrupted after TBI due to high strain forces (Ghajari *et al.*, 2017), is associated with cognitive impairment (Kinnunen *et al.*, 2011; Arenth *et al*., 2014) and is the largest of the commissural fibres. The corticospinal tracts consist of major projection fibres and are associated with motor deficits after TBI (Caeyenberghs *et al.*, 2011; Choi *et al.*, 2012). The corona radiata is a large projection fibre tract and is associated with motor and cognitive impairments after TBI when damaged (Kraus *et al.*, 2007; Caeyenberghs *et al.*, 2011). The inferior longitudinal fasciculi are association tracts in the inferior portion of the brain and are associated with cognitive and functional outcomes after TBI (Edlow *et al.*, 2016). Finally, the middle cerebellar peduncle was selected because of cerebellar atrophy and damage observed after TBI and its association with cognitive and vestibular/balance symptoms after TBI (Drijkoningen *et al.*, 2014; Cole *et al.*, 2018). Importantly, these tracts have varying anatomical orientations, which is likely to be important in assessing diffuse axonal injury because high strain rates are likely to have distinct effects depending on the orientation of the tracts (Parizel *et al.*, 1998; Halabieh and Wan, 2008; Colgan *et al.*, 2010).

### TBI patients demonstrate significant group-level reductions in fractional anisotropy

Comparison of TBI and control data showed significant reductions in FA in patients. A repeated measures ANOVA revealed a significant main effect of group [*F*(1,1960) = 456.57, *P < *0.001] such that TBI patients had lower FA compared to controls. A significant tract × group interaction was also observed [*F*(9,1960) = 15.17, *P < *0.001] with *post hoc t*-tests revealing significantly lower FA in the body, genu and splenium of the corpus callosum, the corona radiata and inferior longitudinal fasciculi in patients (*P < *0.001). Comparison of whole brain mean FA skeleton revealed significantly lower FA in patients compared to controls [*t*(123.4) = 5.84, *P < *0.001]. There were no group level differences in the corticospinal tracts or middle cerebellar peduncle, although there were individuals who showed clearly abnormally low FA in these tracts, illustrating the importance of developing the individual pipeline for diagnosis of axonal injury (Fig. 3B).

### An individual test of white matter damage

**Figure 4:**
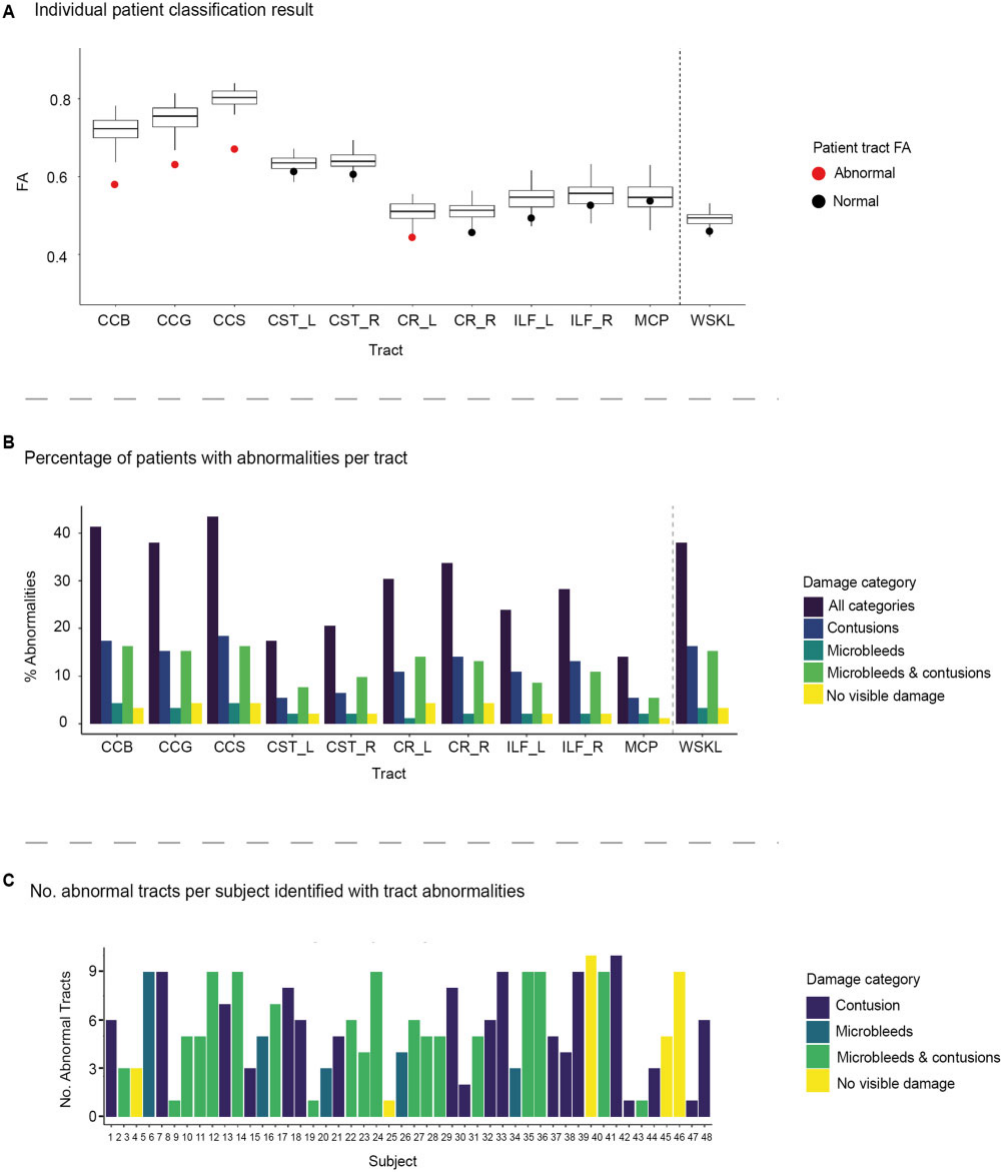
**Diagnostic results using the DTI diagnostic pipeline for each individual TBI patient.** (**A**) Example of individual patient diagnostic results across all tracts and whole brain skeleton. Individual points denote the individual patient FA. Box plots represent the distribution of mean FA for a given tract across all healthy controls (*n = *103). A red circle indicates a significantly abnormal FA value for the individual patient compared to controls (*P *<* *0.05, 95% CI, FDR corrected). A black circle indicates a patient's mean FA value fell within normal ranges compared to healthy controls (*P *<* *0.05, 95% confidence interval, FDR corrected). (**B**) Percentage of patients identified as having an abnormality in tract regions of interest and whole brain skeleton as quantified by diagnostic pipeline *P*-value (two-tailed, 95% CI, FDR corrected). All categories = total percentage of abnormalities across all damage categories. (**C**) Number of tract abnormalities per patient identified as abnormal using diagnostic pipeline compared to 103 healthy controls. Colours represent the damage category of a patient, e.g. yellow indicates where a TBI patient has tract abnormalities but no evidence of damage on routine clinical MRI sequences. CCB/G/S = body/genu/splenium of the corpus callosum; CR_L/R = left/right corona radiata; CST_L/R = left/right corticospinal tract; ILF_L/R = left/right inferior longitudinal fasciculus; MCP = middle cerebellar peduncle; WSKL = whole brain white matter skeleton.

We next applied an individualized approach to identifying axonal injury based on the assessment of whether FA in each patient’s white matter tract was significantly different from the normal range after correction for multiple comparisons. An example of an individual patient’s diagnostic results can be found in Fig. 4A. Highly variable patterns of tract abnormalities were seen in patients. Of the 92 patients individually compared to the healthy control population, 48 patients (52%) were identified as having at least one tract abnormality (e.g. evidence suggestive of axonal injury) compared to the healthy population after FDR correction. Of the patients classified as abnormal, over two-thirds had abnormalities in four or more tracts. Thirty-five patients (38%) were also found to have an abnormal whole brain mean FA compared to healthy individuals with only one patient demonstrating an abnormal whole brain mean FA with no evidence of abnormalities in any tracts. Importantly, a third (∼31%) of patients diagnosed with an abnormality using the pipeline had evidence of tract abnormalities in the absence of whole brain mean FA abnormalities. The body, genu and splenium of the corpus callosum showed the highest rates of abnormalities within the patient population (>35%) with the lowest percentage of abnormalities observed in the middle cerebellar peduncle (15%) (Fig. 4B).

### Diffusion abnormalities in relation to other imaging measure of TBI

We next examined how DTI abnormalities diagnosed using our pipeline related to the presence or absence of other imaging evidence of brain injury (contusion, microbleed, microbleed and contusion or none visible). Patients in all four categories were found to have tract abnormalities (Fig. 4C). Nineteen of 36 patients with contusions (52%) and 19 of 29 patients with both contusions and microbleeds (65%) were found to have tract abnormalities. Five of the 11 patients with only microbleeds (45%) were also identified as having tract abnormalities. Importantly, 5 of 16 patients with no other evidence of brain injury (31%) were identified as having tract abnormality. For the whole brain white matter measure, 14 patients had abnormal whole brain FA measures in patients with both contusion and microbleeds (48%), 15 with contusions (42%), three with microbleeds (27%) and two with no visible damage on MRI (31%).

### Susceptibility weighted imaging and diffusion MRI provide distinct information

Microbleeds have sometimes been used as a surrogate marker of DAI (Scheid *et al.*, 2006), but are actually a marker of diffuse vascular injury. This may co-occur with DAI but can be distinct (Kinnunen *et al.*, 2011; Griffin *et al.*, 2019). Hence, it is important to understand the relationship between DTI and susceptibility weighted imaging (SWI) findings. SWI abnormalities were distinct from diffusion abnormalities as illustrated by the distinct groups of patients identified with or without microbleeds. In one subgroup, patients had microbleed abnormalities (microbleeds or microbleeds and contusions) but no diffusion abnormalities (*n = *16, 40%). A second subgroup had diffusion abnormalities and no microbleed abnormalities (*n = *24, 46%), whilst a third subgroup had both microbleed and diffusion abnormalities (*n = *24, 60%).

### Controlling for the presence of focal lesions

Focal lesions are commonly seen after TBI and can produce diffusion abnormalities, simply because of obvious disruption to white matter structure within a focal lesion. Our main goal is to quantify white matter structure in regions without obvious focal injury. Therefore, our pipeline controls for the presence of focal lesions by excluding them on a case-by-case basis. We investigate the impact of this by calculating FA measures with and without the control of focal injury. Lesion masks were generated for brain injury using T_1_ and FLAIR imaging. These areas were then excluded from the calculation of tract FA and diagnosis performed (see ‘Materials and methods’ section). Sixty-four patients were identified as having a focal contusion/areas of missing brain in a typical distribution for TBI (Supplementary Fig. 2A). Of those patients, 38 were also identified as having an abnormality in at least one tract. Twenty-nine patients with abnormalities in a tract were also found to have abnormal whole brain skeleton FA values when including lesioned areas in our diagnostic pipeline. After exclusion of areas of lesion for each patient and the whole control cohort, 36 of 38 patients remained abnormal. All 29 patients shown to have whole brain FA abnormalities also demonstrated these after exclusion of lesioned areas (Supplementary Fig. 2B). A summary of the diagnostic results with and without correction for lesion can be found in Supplementary Table 4.

### Case studies

**Figure 5:**
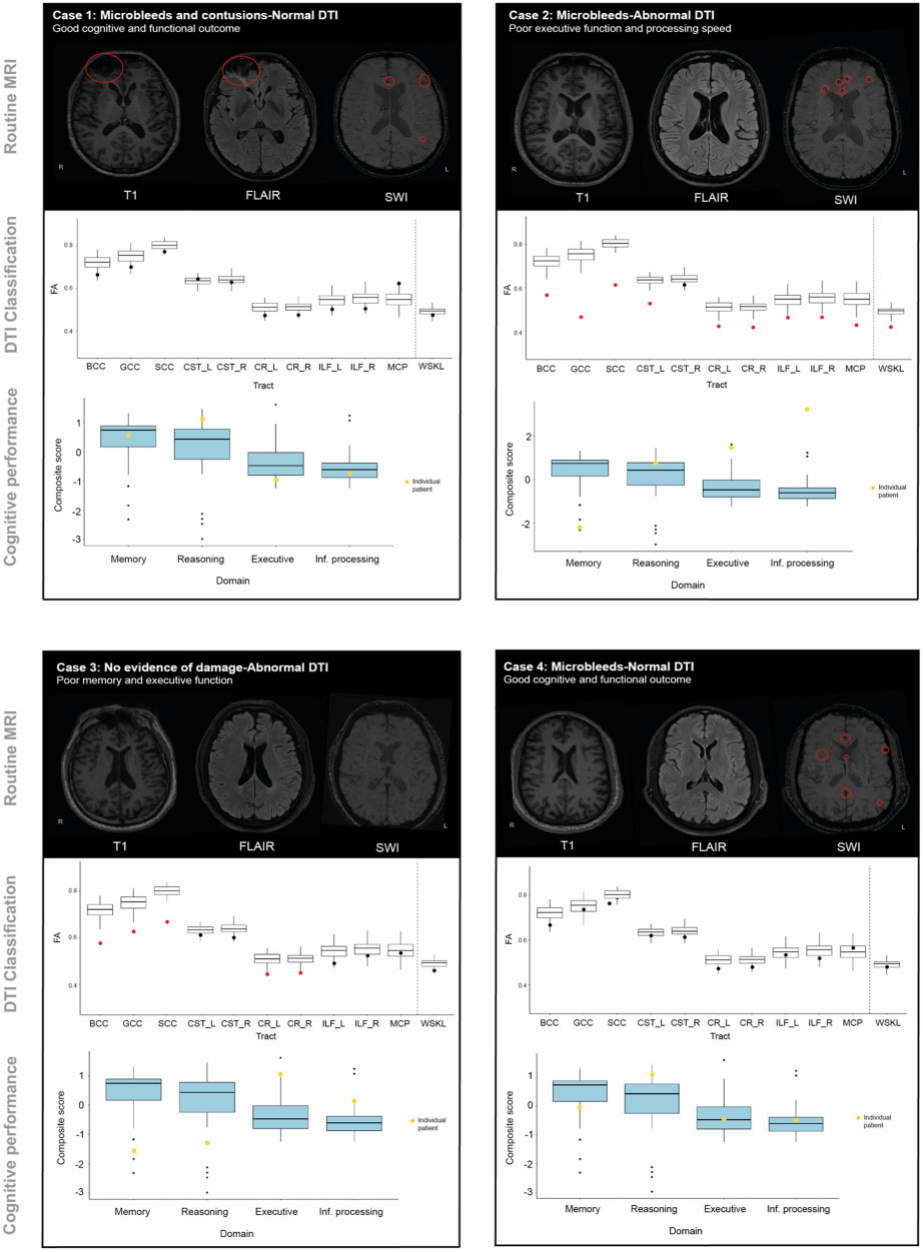
**Individual case studies.** Routine MRI scans including T_1_-MPRAGE, SWI and FLAIR of four moderate-severe TBI patients assessed using the DTI pipeline are observed on the top row of the case study boxes. Areas of visible contusion or microbleeds are highlighted by red circles and confirmed in radiological reports by a consultant neuroradiologist. The *second row* of the case studies box demonstrates the patients individual DTI diagnostic results. Box plots represent FA of the control cohort used in the pipeline (*n = *103). Single points on the plot represents the individual patient assessed with the pipeline. Black dots represent where a patient does not have a significantly abnormal FA within a given region of interest. Red points indicate where patients have a significantly abnormal FA compared to the healthy control cohort, *P *<* *0.05, 95% CI, FDR corrected. *Bottom row* of the case studies illustrate individual patients’ cognitive performance (yellow dot) compared to the control cohort (*n = *35) highlighted as blue box plots. Lower scores in the memory and reasoning domains indicate poorer performance whilst higher scores in the executive and information processing domains indicate poorer performance due to slower reaction times. B/G/SCC = body/genu/splenium of the corpus callosum; CR_L/R = left/right corona radiata; CST_L/R = left/right corticospinal tract; ILF_L/R = left/right inferior longitudinal fasciculus; MCP = middle cerebellar peduncle; WSKL = whole brain white matter skeleton.

The results of the diagnostic pipeline are illustrated with four case studies that highlight how variable DTI findings can be in relation to conventional imaging results, as well as functional, cognitive and psychiatric outcomes (Fig. 5). Case study 1 is a 51-year-old male with moderate-severe TBI as a result of a road traffic accident [lowest Glashow Coma Score (GCS) 3, post-traumatic amnesia (PTA) > 7 days]. Conventional MRI showed evidence of frontal contusions and microbleeds. Upper/moderate disability was present (GOSE = 6) with no severe sleep disturbances (PSQI = 7) but significant depressive symptoms (HADS anxiety = 4, depression *=* 17). Cognitive testing showed performance across cognitive domains to be within normal ranges when compared to healthy individuals (reasoning *z = *0.92, *P > *0.05 memory *z = *0.19, *P > *0.05 executive function *z* = −0.90, *P > *0.05 and information processing *z* = −0.40, *P > *0.05). DTI assessment using the diagnostic pipeline showed no abnormalities

Case study 2 is a 20-year-old male with moderate-severe TBI resulting from of a fall from height (>1 m) onto concrete (lowest GCS 3, PTA 28 days). Conventional MRI showed extensive microbleeds but no focal injury. Upper-moderate disability was present (GOSE = 6), no severe sleep disturbances (PSQI = 6) and no anxiety or depression (HADS anxiety = 1, depression *=* 1). Cognitive testing demonstrated significantly poorer performance on memory (z = −3.14, *P < *0.001), executive function (*z = *2.90, *P < *0.001) and information processing (*z = *7.14, *P < *0.001) compared to healthy controls. Extensive white matter abnormalities were identified in 9 of the 10 tracts as well as abnormalities in whole brain FA.

Case study 3 is a 43-year-old male with moderate/severe TBI resulting from road traffic accident in childhood (∼2 weeks of loss of consciousness, GCS/PTA unknown). Conventional MRI showed no evidence of contusions or microbleeds. There was a good functional recovery (GOSE = 8), no sleep difficulties (PSQI = 4) and no anxiety or depression (HADS anxiety = 0, depression *=* 0). Cognitive impairments in memory (*z* = −2.33, *P = *0.02) and executive function (*z = *2.19, *P = *0.03) were observed when compared to healthy controls. Assessment using the DTI diagnostic pipeline provided evidence of axonal injury in the three subdivisions of the corpus callosum as well as the corona radiata bilaterally.

Case study 4 is a 35-year-old male with moderate/severe TBI resulting from assault with a weapon (loss of consciousness, several minutes, GCS unknown, 2 days PTA). Conventional MRI showed microbleeds and superficial siderosis. There was a good outcome (GOSE = 8), with sleep disturbances (PSQI = 8), and no severe anxiety or depression (HADS, anxiety = 3, depression *=* 0). Performance across all four cognitive domains was within normal ranges when compared to healthy controls (memory *z* = −0.50, *P > *0.05 reasoning *z = *0.91, *P > *0.05 executive *z* = −0.15, *P > *0.05 information processing *z = *0.13, *P > *0.05). Results from the DTI pipeline indicated no evidence of axonal injury in any tract or within whole brain FA.

### Controlling for age in diffusion measurements

Ageing impacts on diffusion MRI measures. Hence, the relationship between FA and age could influence diagnostic results. This is supported by evidence of a significant main effect of age [*F*(1,1010) = 79.7, *P < *0.01] such that FA is lower in older individuals (Supplementary Fig. 3). As expected, a significant main effect of age on whole brain mean FA skeleton was also observed [*F*(1,101) = 18.74, *P < *0.001]. Therefore, we examined the impact of accounting for age when using the diagnostic pipeline. We repeated assessment of the same 92 patients and healthy controls using FA data with the effect of age regressed out. Both age and age^2^ was regressed out of FA using multiple linear regression and the residuals were then used in the diagnostic pipeline. As with non-age regressed data, the same 48 patients (52%) were identified as having at least one abnormal tract compared to healthy controls (Supplementary Fig. 4). A summary of individual diagnostic results with and without age correction can be found in Supplementary Table 5.

### Control sample size influences specificity of diffusion diagnostic pipeline

**Figure 6:**
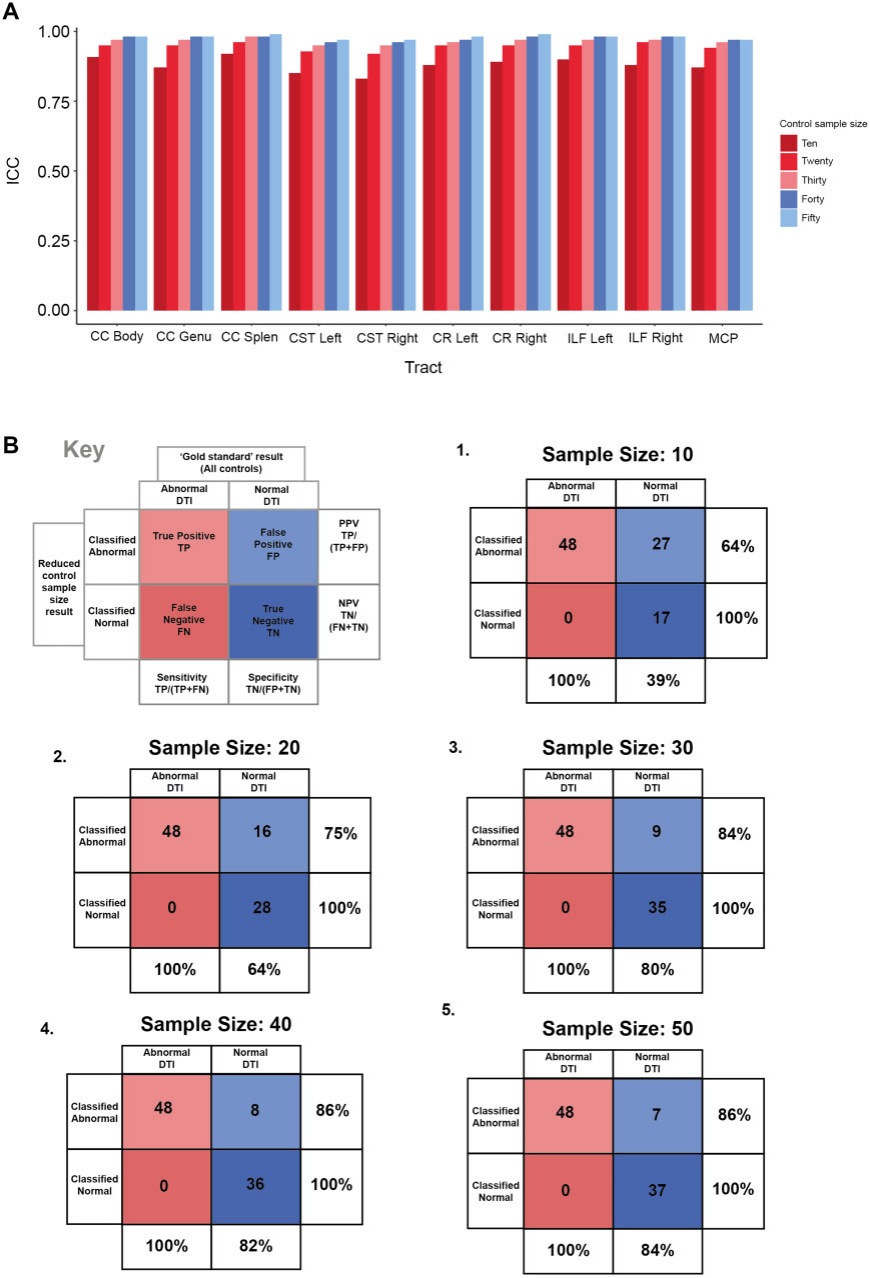
**Reliability and accuracy measures using smaller control group sample sizes to identify DTI abnormalities in individual TBI patients.** (**A**) ICC values derived from the FDR corrected *P*-values of individual patient’s tract FA when compared to randomly subsampled control groups of a given *n* over 100 iterations. Sample sizes ranged from 10 to 50 and were randomly selected from the total control cohort (*n = *103). (**B**) Calculation of type 1 and type 2 errors and related measures of sensitivity, specificity, positive and negative predictive value for diagnostic rates of individual TBI patients when compared to a control group of a given *n* (e.g. 10–50 in increments of 10). Values are calculated by comparing results of smaller control group to the results derived for each patient when compared to all 103 healthy controls. CC = corpus callosum; CR = corona radiata; CST = corticospinal tract; ILF = inferior longitudinal fasciculus; MCP = middle cerebellar peduncle.

One important practical consideration is the size of a control group collected on the same scanner that is necessary to produce stable performance of axonal injury diagnosis. To investigate this, we examined diagnostic rates of patients (e.g. normal or abnormal tracts) across a range of control group sample sizes (10, 20, 30, 40 and 50). ICCs were calculated for tract corrected *P*-values derived for each patient across 100 iterations for each sample size (Fig. 6A). High ICC was observed for all tracts and sample sizes (e.g. >0.67); however, smaller sample sizes demonstrated the lowest ICC indicating lower reliability.

To assess the impact of control group sample size on sensitivity and specificity, type 1 and type 2 errors were quantified (Fig. 6B). When compared to the diagnostic rates using the whole control cohort, no false negatives were observed across any of the sample size results i.e. no patient was diagnosed as having normal DTI in smaller control group sample size tests when also diagnosed as having abnormal DTI using the whole control cohort. Similarly, all patients diagnosed as abnormal using the whole control group, were also found to be diagnosed as abnormal across all smaller sample size tests, demonstrating 100% sensitivity. In contrast, higher rates of false positives (patients deemed abnormal in smaller sample sizes in contrast to the whole control group) were observed when a smaller control group sample size was used. The smallest sample size of 10 identified 27 additional patients leading to a 39% specificity rate. Specificity more than doubled when using a control sample size of 30 (specificity = 80%) and only incrementally improved when using 40 (82%) or 50 healthy controls (84%) to diagnose DTI abnormalities in individual patients. Similarly, the positive predictive value increased by 20% between sample size of 10 to 30, only incrementally increasing with sample sizes >30.

### High specificity for identifying abnormalities in TBI patients compared to healthy controls

To determine whether the methods applied are specific to TBI, we applied the same diagnostic method to each individual control, who were compared to the rest of control group. Of the 103 healthy controls individually assessed against the remaining control cohort, three (2.9%) were found to have a single abnormal tract using our diagnostic pipeline. Six controls were found to have an abnormal whole brain skeleton (5.8%) when individually compared to the remaining control cohort. These rates of false positives are within the expected statistical range.

### Functional and cognitive impairments are greater in patients diagnosed with axonal injury when using the pipeline

Groups with and without white matter abnormalities as diagnosed using the pipeline were compared across functional, cognitive and psychiatric outcomes. TBI patients in general showed a typical pattern of cognitive impairment. Impairments were seen in memory, processing speed and executive functioning after FDR correction (Supplementary Table 6). Specifically, TBI patients performed significantly poorer on the People’s Test [immediate *t*(64.39) = 2.84, *P < *0.01; and delayed recall *t*(63.07) = 2.39, *P < *0.02], TMT A [*t*(111) = −4.89, *P < *0.001], TMT B [*t*(115) = −4.44, *P < *0.001] and TMT A − B [*t*(109.7) = −3.36, *P < *0.01] as well as subscores of the DKEFS Stroop test [colour naming: *t*(90.18) = −5.67, *P < *0.001; word reading *t*(79.19) = −4.65, *P < *0.001; and inhibition switch *t*(101) = −3.69, *P < *0.001], FDR corrected.

**Figure 7:**
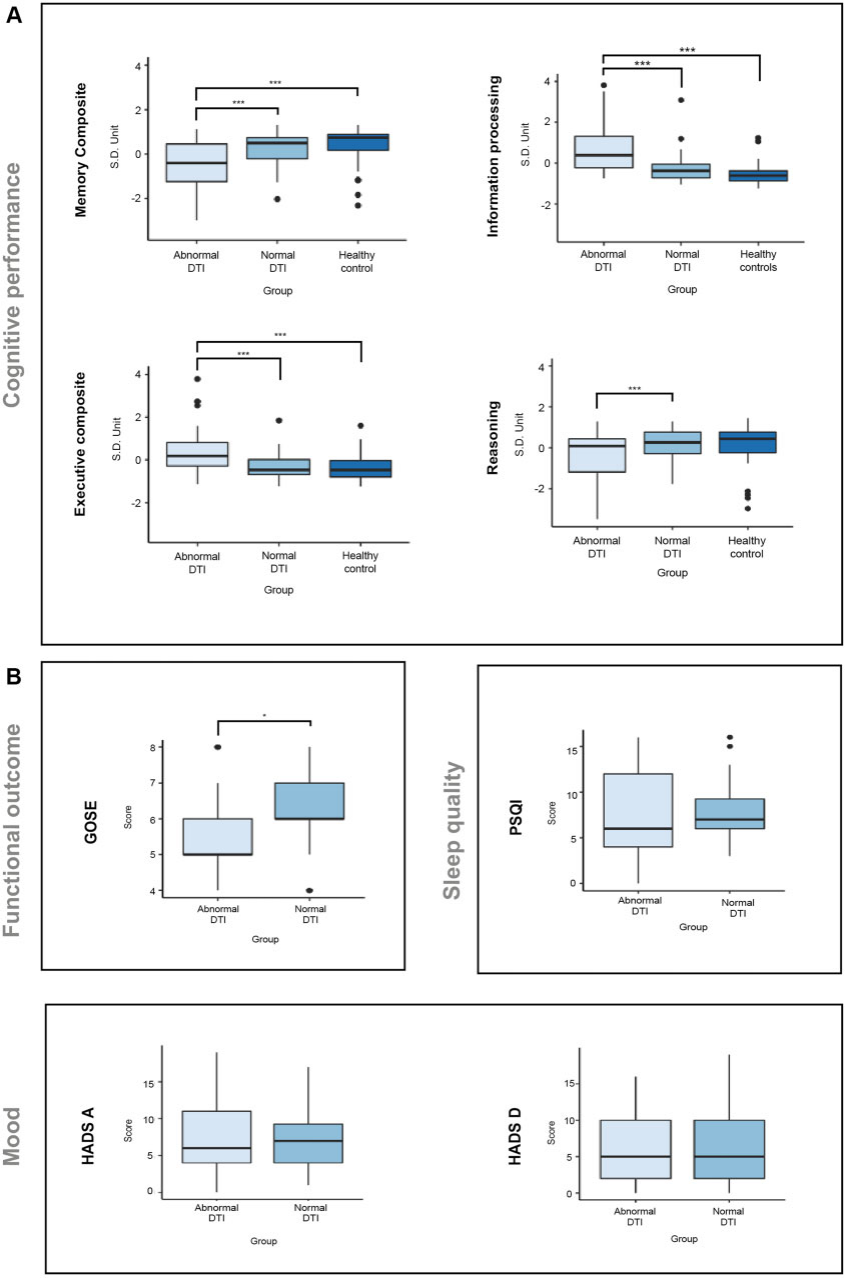
**Reliability of diagnostic pipeline across varying control sample sizes.** (**A**) Comparison of composite scores for the four cognitive domains selected between healthy controls and patients identified as having either normal or abnormal DTI using the DTI pipeline. Diagnosis of normal versus abnormal DTI was determined using the whole control cohort (*n = *103), not accounting for age. (**B**) Comparison of functional outcome measures between patients identified as having normal versus abnormal DTI using the diagnostic pipeline. ***P <* *0.001, *P <* *0.05.

Patients with evidence of axonal injury, as diagnosed using our pipeline, were more impaired across all cognitive domains compared to patients without DTI abnormalities. For memory, a repeated measures ANOVA including group and age revealed a significant main effect of group [*F*(2,124) = 13.147, *P < *0.001]. *Post hoc* Tukey *t*-tests showed this effect was driven by significantly lower scores for patients diagnosed with tract abnormalities using the pipeline compared to those diagnosed with normal DTI (*P < *0.001) and controls (*P < *0.0001). There was no significant difference between controls and patients with normal DTI (*P = *0.66) (Fig. 7A). There was no main effect of age or interaction of age with group on memory scores. For executive function, ANOVA showed a main effect of group [*F*(2,124) = 17.088, *P < *0.001]. This was driven by slower performance in patients diagnosed with tract abnormalities using the pipeline, relative to patients with normal DTI (*P < *0.001) and healthy controls (*P < *0.001). A main effect of age was also observed [*F*(1,124) = 6.55, *P < *0.05] but no interaction between group and ae (*P = *0.138). For information processing, there was a significant main effect of group [*F*(2,124) = 24.524, *P < *0.001], driven by patients diagnosed as having abnormal DTI using the pipeline performing more slowly than patients with normal DTI (*P < *0.001) and controls (*P < *0.001). There was no difference between patients diagnosed with normal DTI and controls (*P = *0.230). There was no main effect of age nor an interaction with group. A significant main effect of group [*F*(2,124) = 4.463, *P < *0.05] was also observed when performing a repeated measures ANOVA for reasoning scores, driven by lower performance in patients diagnosed with abnormal DTI using the pipeline compared to patients without DTI abnormalities (*P = *0.029). However, there was no significant difference between patients diagnosed as abnormal and controls (*P = *0.051) and no difference between patients diagnosed with normal DTI and controls (*P = *0.98). There was a significant main effect of age [*F*(1,124) = 7.99, *P < *0.01]; however, there was no interaction between group and age.

The GOSE provided a summary measure of clinical outcomes. Patients diagnosed with abnormal DTI using the pipeline showed lower scores on the GOSE than patients with normal DTI [*t*(80.65) = 2.59, *P < *0.02] (Fig. 7B). There were no significant differences between patient groups on measures of sleep disturbance (PSQI) or measures of psychiatric state provided by the anxiety and depression subscales of the HADS questionnaires (*P > *0.05) (Fig. 7C).

We also investigated whether variable patterns of DTI abnormality in different tracts related to cognitive performance. Bivariate correlations were performed between all 10 tracts and each of the cognitive composite measures, correcting for multiple comparisons within each cognitive domain. For executive function, significant negative correlations were observed in the body (*P < *0.004), genu (*P < *0.0.001), splenium (*P < *0.001), left and right inferior longitudinal fasciculi (*P < *0.004 and *P < *0.001, respectively) and middle cerebellar peduncle (*P < *0.01), (Supplementary Fig. 5). For memory, positive correlations with the genu of the corpus callosum were seen (*P < *0.05) (Supplementary Fig. 6). For information processing speed, negative correlations with FA in the corpus callosum body (*P < *0.001), genu (*P < *0.001), splenium (*P < *0.001), right corona radiata (*P < *0.05), left and right inferior longitudinal fasciculus (*P < *0.01 and *P < *0.001, respectively) and middle cerebellar peduncle (*P < *0.001) were observed (Supplementary Fig. 7). Finally, correlations between reasoning and tract FA were observed in the corpus callosum body (*P < *0.01), genu (*P < *0.01) and splenium (*P < *0.05) as well as the left and right inferior longitudinal fasciculi (*P < *0.05 and *P < *0.01, respectively) as well as the left and right corona radiata (*P < *0.01 and *P < *0.01, respectively) and middle cerebellar peduncle (*P < *0.01) (Supplementary Fig. 8).

### Functional and cognitive impairments do not differ between patients when categorized based on visible damage

To assess whether the cognitive and functional differences observed between patients diagnosed with or without diffusion abnormalities are specific to axonal injury, patients were also compared based on visible damage on routine MRI. For example, patients with or without microbleeds and patients with or without focal lesions were compared across the four cognitive domains and GOSE. When grouping patients by microbleeds (*n = *40) versus no microbleeds (*n = *52), cognitive performance did not significantly differ between the groups for any of the four cognitive domains (*P > *0.1, FDR corrected) (Supplementary Fig. 9). Similarly, no significant difference was observed between patients for GOSE scores [*t*(61.36) = 0.16, *P = *0.87] (Supplementary Fig. 10). When comparing cognitive performance between patients with (*n = *65) or without (*n = *27) focal lesions (Supplementary Fig. 11), no significant differences were observed across any of the cognitive domains (*P > *0.05, FDR corrected). In addition, no significant difference was observed between GOSE scores [*t*(27.87) = −0.20, *P = *0.84] (Supplementary Fig. 12).

### Greater intervals between injury and MRI are observed in patients diagnosed with axonal injury when using the pipeline

The relationship between time since injury and diagnosis of axonal injury using our pipeline was explored (Supplementary Fig. 13). Patients identified with axonal injury had significantly greater time since injury than patients without [*t*(86.14) = 3.13, *P < *0.01], although widespread intervals were observed in both groups (group with tract abnormalities, range 6–441 months; group without abnormalities, range 6–497 months since injury). There was no significant correlation between the number of tracts identified as abnormal and time since injury in patients with axonal injury (*r = *0.17, *P = *0.26). Bivariate correlations between individual tract FA and time since injury revealed significant relationships (*P < *0.05, FDR corrected) in the body (*r =* −0.38), genu (*r =* −0.38) and splenium of the corpus callosum (*r =* −0.43) as well as the right corona radiata (*r =* −0.24), and middle cerebellar peduncle (*r =* −0.27) such that the greater the time since injury the lower FA. When correlating time since injury with whole brain mean FA, a significant negative correlation was also observed (*r =* −0.37, *P < *0.001).

### Diagnosis of axonal injury in subacute TBI patients is reliable but longitudinal assessments are recommended

**Figure 8:**
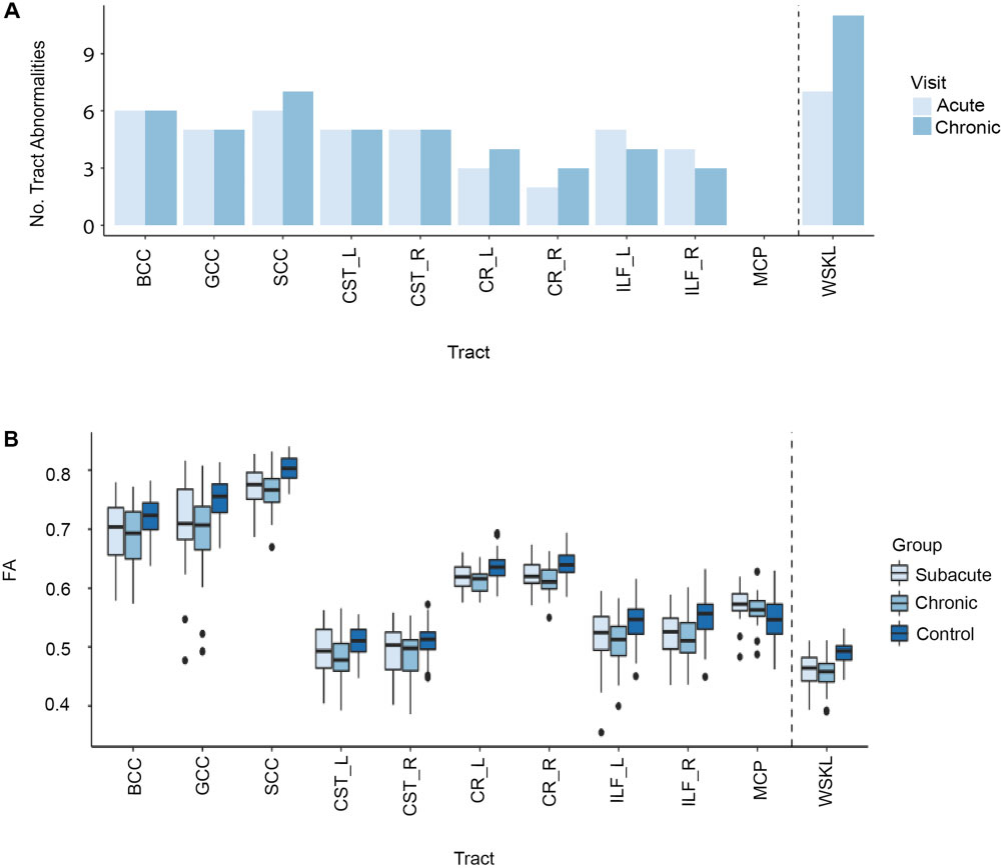
**Longitudinal diagnostic results of subacute TBI cohort (*n = *25).** (**A**) Number of abnormal tracts identified in longitudinal subacute TBI cohort using diagnostic pipeline for subacute and chronic time points. (**B**) Tract fractional anisotropy measures from subacute (10 days to 6 weeks) and chronic (∼6 months) time points. B/G/SCC = body/genu/splenium of the corpus callosum; CR_L/R = left/right corona radiata; CST_L/R = left/right corticospinal tract; ILF_L/R = left/right inferior longitudinal fasciculus; MCP = middle cerebellar peduncle; WSKL = whole brain white matter skeleton.

We examined whether the application of our pipeline to a subacute TBI cohort was able to reliably identify axonal injury. At the individual level, 7 of 25 patients were found to have an abnormality (28%). Of those patients, six had abnormalities in at least one tract in addition to the whole brain skeleton, whilst an additional patient showed only abnormalities in the whole brain skeleton. When assessed at the chronic time point (∼6 months), 11 patients (44%) had abnormalities. All seven patients with subacute abnormalities remained abnormal at 6 months, with the same tracts and/or whole brain skeleton abnormalities being observed across the two time points (Fig. 8A). A repeated measures ANOVA revealed no significant main effect of time point on the number of abnormalities observed across tracts [*F*(1,18) = 0.05, *P = *0.9]. Of the four additional patients diagnosed with axonal injury at the chronic time point, one had tract abnormalities and the remaining three demonstrated abnormalities in the whole brain skeleton (Fig. 8B). Calculation of the intraclass correlation coefficient of *Z*-scores derived from the diagnostic pipeline revealed good reliability across tracts (mean ICC = 0.89, range 0.77–0.96) between the two time points. When examining the ICC of *P*-values (FDR corrected) derived from the diagnostic pipeline between the two time points (Supplementary Fig. 14), average reliability remained high, although greater variability was observed across tracts, likely reflecting the changes in FA longitudinally (mean ICC = 0.76, range 0.59–0.86). Examination of ICC for whole brain mean skeleton between acute and chronic time points revealed high reliability for *Z*-scores (ICC = 0.92), and good reliability for *P*-values (FDR corrected) derived from the diagnostic pipeline (ICC = 0.70).

At the group level, there was evidence of reductions in FA in patients at both subacute and chronic time points compared to controls (Fig. 8B). In subacute scans, a factorial ANOVA revealed a significant main effect of group [*F*(1,1270) = 79.195, *P < *0.001], such that patients had significantly lower FA than healthy controls. A significant group × tract interaction was also observed [*F*(9,1270) = 5.534, *P < *0.001] with *post hoc* Tukey tests revealing that this interaction was driven by significantly lower FA in the genu (*P *<* *0.001) and splenium (*P < *0.01) of the corpus callosum as well as the left and right inferior longitudinal fasciculi (*P < *0.02, respectively). Comparison of whole brain skeletonized FA revealed significantly lower FA in patients at the subacute time point compared to controls [*t*(30.27) = −4.66, *P < *0.001]. When comparing FA at the 6-month follow-up to healthy controls, factorial ANOVA revealed a significant main effect of group [*F*(1,1270) = 171.49, *P < *0.001] such that patients had lower FA than controls, as well as a significant group × tract interaction [*F*(9,1270) = 6.36, *P < *0.001]. *Post hoc* Tukey tests revealed that this interaction was driven by significantly lower FA in the body (*P < *0.001), genu (*P < *0.001) and splenium (*P < *0.001) of the corpus callosum as well as the left and right corona radiata (*P < *0.01 and *P < *0.02, respectively), right corticospinal (*P < *0.05) and left and right inferior longitudinal fasciculi (*P < *0.001, respectively). Comparison of whole brain skeleton FA also revealed a significant reduction in patients at the 6-month follow up compared to controls [*t*(30.27) = −5.70, *P < *0.001].

When comparing patients between the two time points, repeated measures ANOVA revealed a significant main effect of time [*F*(1,500) = 5.619, *P = *0.02] such that lower FA was observed in the chronic compared to subacute phase of injury across all tracts (Fig. 8A). No significant tract × time interaction was observed. No significant difference was observed for whole brain skeletonized mean FA between the two time points [*t*(49.95) = 0.88, *P = *0.39].

## Discussion

Our work shows that DTI can be informative for evaluating white matter structure in individual patients. We describe a robust approach to the calculation of FA that controls for a number of important methodological constraints. Diffusion MRI abnormalities indicative of DAI are common in the chronic phase after moderate/severe TBI (∼50% of chronic and ∼30% subacute patients in our sample) and we show that they provide distinct information from other commonly used diagnostic approaches. The pipeline can be used to detect white matter abnormalities in the subacute stage (>10 days after injury) and is sensitive to changes in FA that develop in the first months after injury. The location of DAI in the brain is variable, so an individualized tract-based assessment method is necessary. Patients may show abnormalities in tracts and not the whole brain skeleton (∼31% of those diagnosed with an abnormality), demonstrating the importance of investigating specific tracts. Importantly, some patients without visible damage using more standard MRI, including SWI, show clear evidence of diffuse axonal injury on diffusion measures. Conversely, patients may show SWI evidence of diffuse vascular injury in the absence of diffusion abnormalities. Hence, diffusion and SWI MRI provide complementary information in the assessment of white matter abnormalities after TBI. In addition, patients with evidence of DAI as determined using our pipeline, show cognitive impairments and poorer functional outcomes which cannot be attributed to psychiatric difference. We therefore provide a framework and methodology for the use of diffusion MRI in the assessment of DAI that provides clinically useful information in individual patients.

Several measurements can be calculated from diffusion MRI. We focus on FA. This provides a measure of white matter tract integrity that has been widely studied in previous TBI work (Mac Donald *et al*., 2007*b*; Sidaros *et al.*, 2008). FA calculations are based on patterns of water diffusion that are normally highly directional in much of the white matter because of the directional organization of axons (Mori and Zhang, 2006). Mechanical forces applied to white matter tracts at the time of an injury damage the normal axonal structure producing more random patterns of water diffusion along a tract (Song *et al.*, 2002; Budde *et al.*, 2011). Alterations in FA have been validated as a measure of axonal injury in animal models of TBI, with reductions in FA associated with disrupted white matter structure and the associated inflammatory response (Mac Donald *et al.*, 2007*a*, *b*; Laitinen *et al.*, 2015).

We extend previous studies by demonstrating a robust method for measuring white matter tract FA at the individual level. We and many others have previously shown the value of diffusion MRI in assessing patterns of white matter injury in groups of TBI patients. Widespread evidence of DAI is almost always present when moderate/severe TBI patients are studied. The location and severity of injury relates to patterns of cognitive and functional outcomes (Sidaros *et al.*, 2008; Kumar *et al.*, 2009; Kinnunen *et al.*, 2011; Bonnelle *et al.*, 2012). However, the significant inter-individual heterogeneity in the location and severity of DAI motivates the need for individualized assessments. Here we show that ∼52% of moderate/severe TBI patients have diffusion abnormalities indicative of DAI and that these abnormalities are often missed using T_1_, FLAIR or SWI imaging. Hence, clinicians will often be falsely reassured about the absence of DAI using standard neuroimaging investigations.

We show that diffusion MRI provides information that is distinct from other assessments of post-traumatic injury. DAI can occur in the absence of focal brain injury, which we see in the subgroup of patients who have diffusion abnormalities in the absence of any evidence of focal injury. In addition, we confirm that diffusion MRI provides complementary information to SWI, which identifies microbleeds. Although SWI has previously been thought of as a surrogate marker of DAI (Scheid *et al.*, 2006), recent work confirms that microbleeds are a marker of diffuse vascular injury (Costello *et al.*, 2013; Liu *et al.*, 2014; Griffin *et al.*, 2019). Importantly, DAI can occur with or without diffuse vascular injury (Liu *et al.*, 2014; Griffin *et al.*, 2019). We demonstrate that only 45% of patients with microbleeds were found to have evidence of axonal injury. Conversely, more than a third of patients with no visible damage on conventional MRI had evidence of axonal injury. In a clinical setting, diagnosis may be made based on microbleeds where no axonal injury is present or where an unremarkable MRI leads to diagnosis being determined on clinical symptoms which may not be reliable. Our findings provide evidence of these potential pitfalls in the current clinical diagnosis of DAI and illustrate the benefits of a DTI diagnostic pipeline, particularly when observing behavioural impairments in patients with unremarkable MRI’s.

Diffuse axonal injury is an important predictor of cognitive and functional outcomes (Katz and Alexander, 1994; Scheid *et al.*, 2006; Weiss *et al*., 2007; Menon and Zahed, 2009). We show that individuals with evidence of DAI show more cognitive impairments in all four cognitive domains assessed. In contrast, patients without evidence of DAI showed no cognitive abnormalities in comparison to healthy controls. Patients with evidence of DAI had significantly poorer functional outcomes, emphasizing the importance of DAI for real-world clinical outcomes. Differences in cognitive and functional outcomes between these two patient groups were not obviously attributable to mood, as patients with and without evidence of DAI showed similar levels of depressive and anxiety symptomatology. Importantly, the presence of focal injury and diffuse vascular injury was not associated with worse cognitive or functional outcomes, showing that diffusion MRI provides specific information about that is related to chronic impairments after TBI that is not provided by more conventional neuroimaging.

Patterns of white matter damage vary across individuals, which reflects the variable biomechanics of different head injuries. Tract damage is produced by large forces exerted at the time of an injury and the location of these forces is highly dependent on the specific features of a head impact (Ghajari *et al.*, 2017), with rotational forces having differential effects depending on the direction of the axons and force (Halabieh and Wan, 2008; Colgan *et al.*, 2010). Hence, it is necessary to estimate injury from a range of different tracts. We describe a principled selection process for choosing the final set of tracts we study. These tracts are large enough to reliably estimate white matter structure (Sairanen *et al.*, 2017). They have good test-retest reliability, which we demonstrate in data from healthy controls scanned multiple times over a 2-year period. They were selected to have different anatomical orientations so the impact of a range of biomechanical forces can be assessed (Parizel *et al.*, 1998; Halabieh and Wan, 2008; Colgan *et al.*, 2010), and also to include association, projection and commissural pathways so the impact of DAI on varying types of brain networks can be measured.

The distribution of white matter tract damage is also known to relate to patterns of cognitive impairment (Kinnunen *et al.*, 2011; Bonnelle *et al.*, 2012; Palacios *et al.*, 2012). Hence, the distribution of damage to specific tracts may explain patterns of post-traumatic impairments. This principle has previously been demonstrated by the association of corticospinal tract damage to motor deficits (Caeyenberghs *et al.*, 2011; Choi *et al.*, 2012). Here, we report clear correlations between diffusion abnormalities and a range of cognitive impairments for several the tracts studied. For example, significant negative correlations were observed between executive function and the three subdivisions of the corpus callosum, the inferior longitudinal fasciculi, the middle cerebellar peduncle and executive function. In addition, when examining information processing and reasoning, significant correlations were observed with the three subdivisions of the corpus callosum, the corona radiata bilaterally, the inferior longitudinal fasciculi and the middle cerebellar at varying degrees.

We have considered a range of potential limitations for the use of diffusion MRI in this context and we address a number of practical issues relevant to the widespread adoption of diffusion MRI for the diagnosis of DAI in clinical practice. One barrier is the need to compare individual diffusion measures to control data collected on the same scanner. Currently, this is necessary because the between-scanner variability of diffusion measures is high enough to impact in an unpredictable way on patient classification. Hence, an important consideration is what is the minimum size of a control group necessary for adequate sensitivity and specificity. We explore this issue by bootstrapping the diagnostic analysis using different control groups of differing sizes. We conclude that a control sample size of around 30 with a range of ages provides acceptable ‘specificity’ and ‘sensitivity’. We would therefore recommend that clinical units collect around 30 control subjects using the same diffusion sequence parameters to form the comparative dataset for the DAI analysis pipeline we provide. This recommendation may change in the future as recent work indicates that diffusion data can be harmonized across scanners using groups of around 15 control subjects to identify the scanner dependent variability from the diffusion signal prior to analysis (Karayumak *et al.*, 2019).

Another factor that should be considered when comparing FA across individuals is their age. There is a non-linear relationship between age and FA which could impact on the identification of diffusion abnormalities (Pfefferbaum *et al.*, 2000). We explicitly investigated this issue by comparing diagnostic results before and after statistically controlling for the age of the patients and the comparative control group. Regressing age and age^2^ out of the patient and control data had little impact on the diagnostic result i.e. the same patients were identified as having evidence of DAI with and without when accounting for age. Nevertheless, we recommend the use of age-regression when using the pipeline as this might correct differences in age between other control cohorts and patient groups. Hence, our method allows for the inclusion of healthy individuals somewhat older or younger than the patient without biasing the results, which may prove useful in clinical settings where data are sparse.

The presence of focal lesions also potentially impacts on the reliability of FA estimates. This is an important consideration when assessing DAI in individual patients as the mean FA across a tract is calculated and may therefore be biased if a portion is missing. We provide a solution to this issue by excluding areas of the focal lesion from white matter tract estimating in both the patient and controls. This has a small effect on the diagnostic results, with 3 of 38 patients no longer found to have evidence of axonal injury. These findings illustrate the specificity of the pipeline in primarily detecting axonal injury but also highlights that it is possible for large lesions to bias results if not accounted for. We therefore recommend excluding areas of focal lesion/missing brain from the pipeline to accurately quantify DAI.

Finally, it is important to consider the timing of diffusion MRI acquisition relative to the injury. We have evaluated our pipeline for use in the assessment of both chronic and subacute moderate-severe TBI. TBI produces dynamic changes in diffusion measures including increases in FA in the initial days (Mac Donald *et al.*, 2007*a*), and progressive reductions many years after injury. In our chronic TBI population, patients identified with axonal injury had significantly greater intervals between their date of injury and MRI, than those without. Furthermore, significant negative relationships between FA and time since injury were observed in several tracts. In addition, FA abnormalities became more pronounced within individuals from the subacute phase at their chronic phase. These findings suggest that FA abnormalities become more prominent with time since injury, which is most likely due to a progressive neurodegenerative process that is triggered by TBI (Graham and Sharp, 2019). Further application of this method in a larger acute population would be beneficial to determine whether diagnosis acutely corresponds to cognitive and functional outcomes within the chronic phase.

In summary, we describe a pipeline for diffusion MRI assessment of diffuse axonal injury in individual patients and provide data and code for use by others. We show that this is a robust approach to the calculation of FA that controls for a number of important methodological constraints. Diffusion MRI abnormalities indicative of DAI are common in the subacute and chronic phase after moderate/severe TBI and provide diagnostic information that is distinct from other commonly used neuroimaging approaches that is clinically valuable.

## Supplementary Material

awaa372_Supplementary_Data

## Abbreviations

DAI: diffuse axonal injury
DTI: diffusion tensor imaging
FA: fractional anisotropy
GOSE: Glasgow Outcome Scale-Extended
HADS: Hospital Anxiety And Depression Scale
PSQI: Pittsburgh Quality of Sleep Index
SWI: susceptibility weighted imaging
TBI: traumatic brain injury
