# Supplementary material for: Detecting axonal injury in individual patients after traumatic brain injury
Source: Brain. 2020 Nov 30;144(1):92–113. doi: 10.1093/brain/awaa372 (PMC7880666; doi:10.1093/brain/awaa372)
Supplement: awaa372_Supplementary_Data [file awaa372_supplementary_data.pdf]

Supplementary material

Supplementary Figures

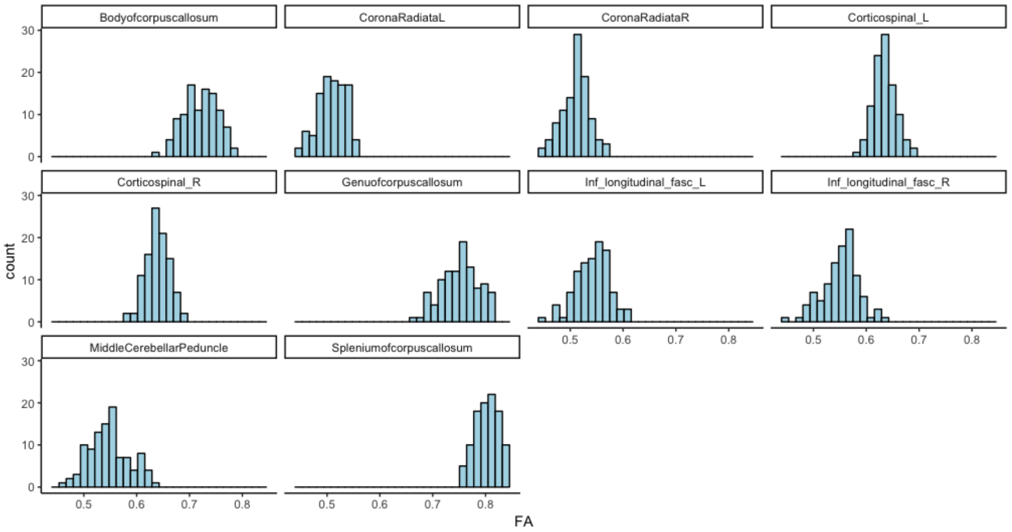

Supplementary figure 1: Distribution of FA in healthy control cohort (n=103) across all 10 tracts selected for use in the DTI diagnostic pipeline.

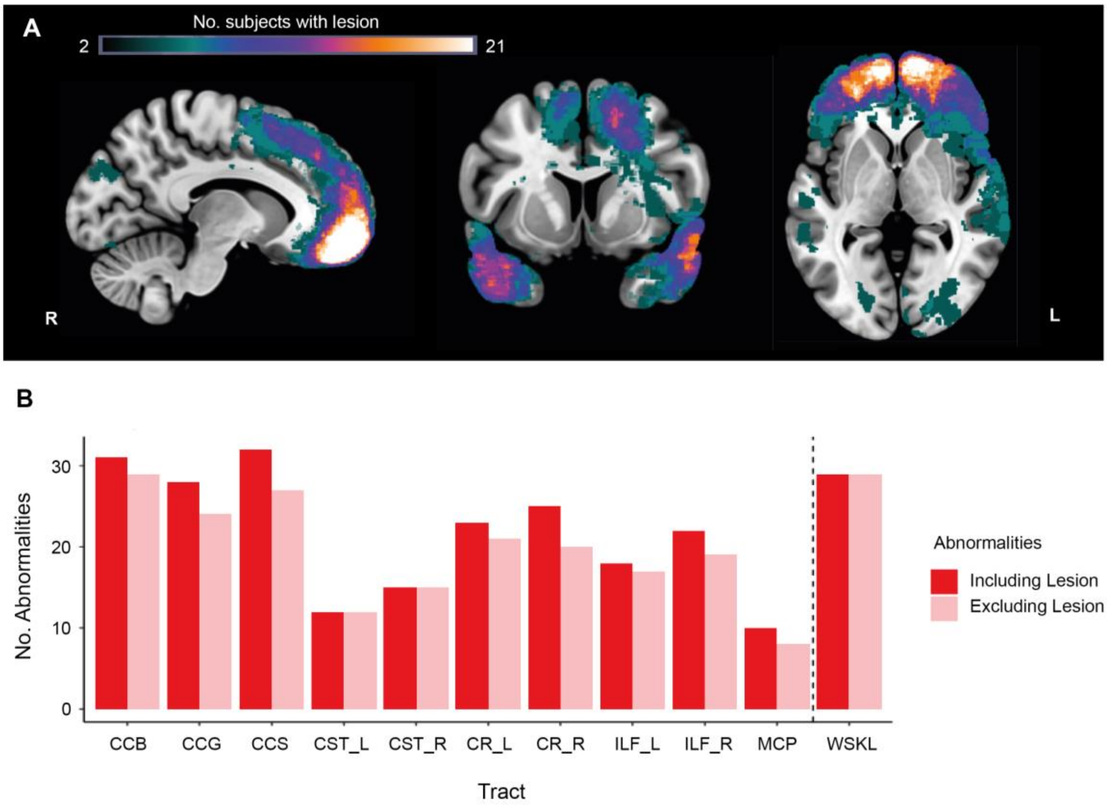

Supplementary figure 2: Diagnostic results when including or excluding areas of lesion. A. Lesion overlap map of the 64 patients identified as having a contusion/area of missing brain. Lesion masks are averaged across patients to demonstrate areas of high overlap (bright white) and areas of low overlap (dark green) across patients. **B.** Tract abnormality rates within the 64 patients when including lesioned areas (red) and when excluding areas of lesion (pink).

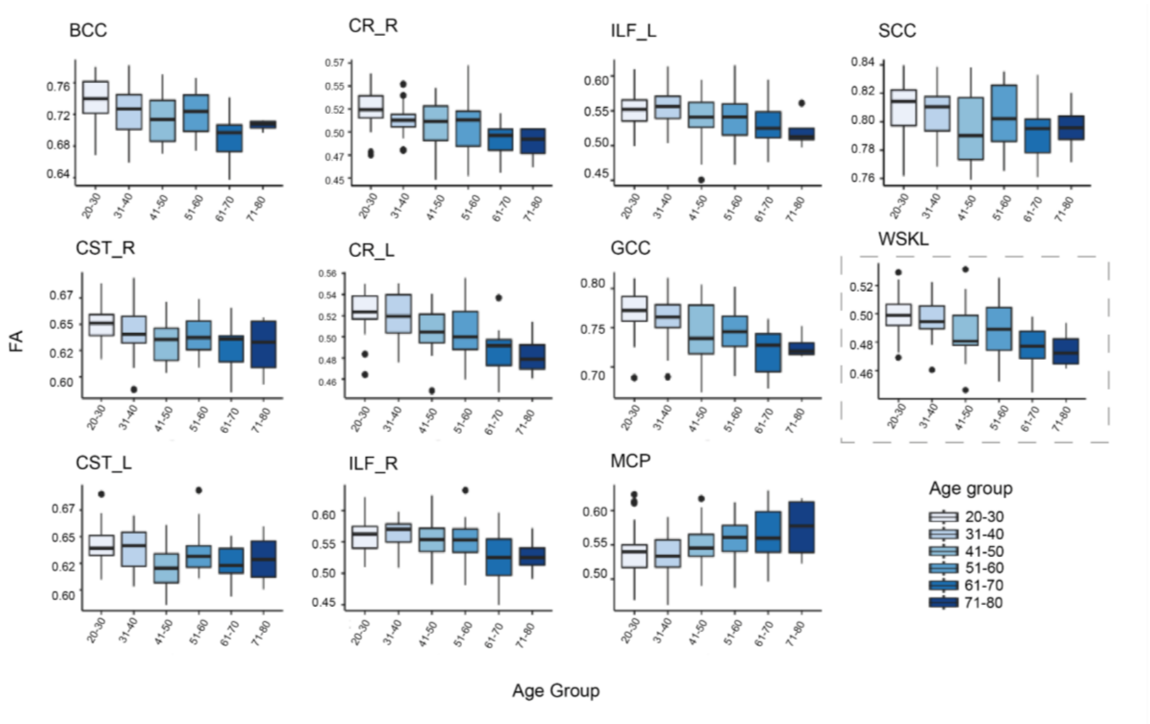

Supplementary figure 3: Tract mean FA for healthy control cohort (n=103), grouped by ages ranging from 20-30 years to 71-80 years. BCC=Body of corpus callosum, CR_R= right Corona Radiata, ILF_L=Left inferior longitudinal fasciculus, SCC=Splenium of corpus callosum, CST_R=right corticospinal tract, CR_L= left corona radiata, GCC=Genu of corpus callosum, WSKL=Whole brain white matter skeleton, CST_L=left Corticospinal tract, ILF_R=right inferior longitudinal fasciculus, MCP=Middle cerebellar peduncle.

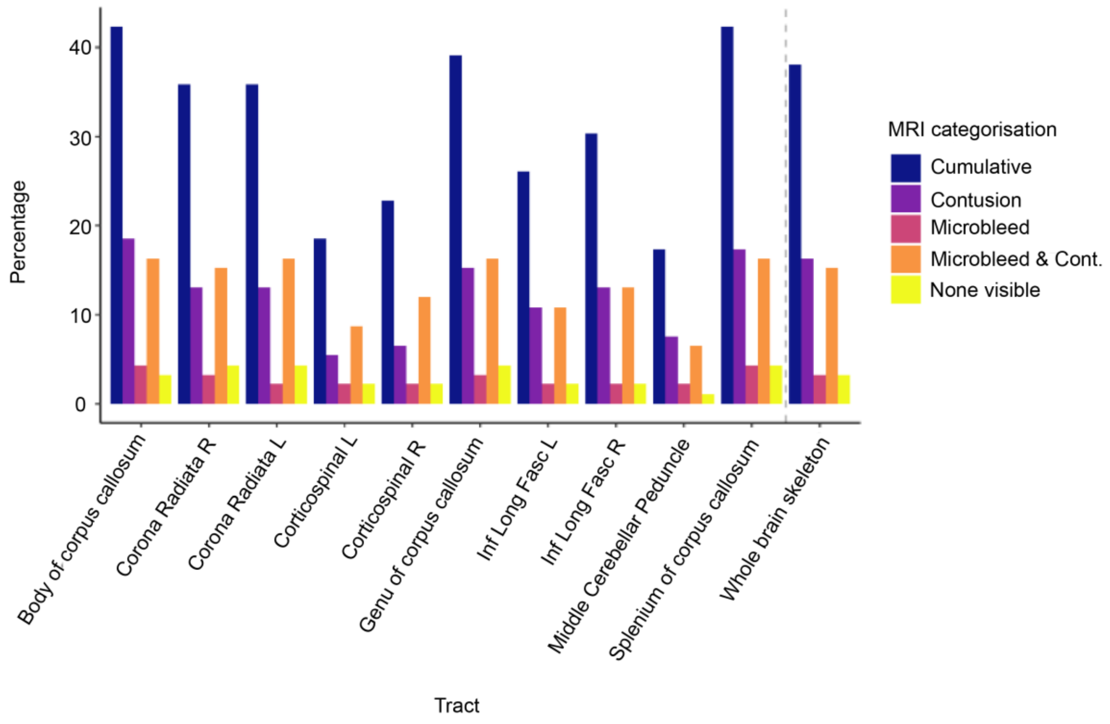

Supplementary figure 4: Tract ROI diagnostic rates (%) in TBI patients when regressing age out of FA data with healthy controls cohort (n=103).

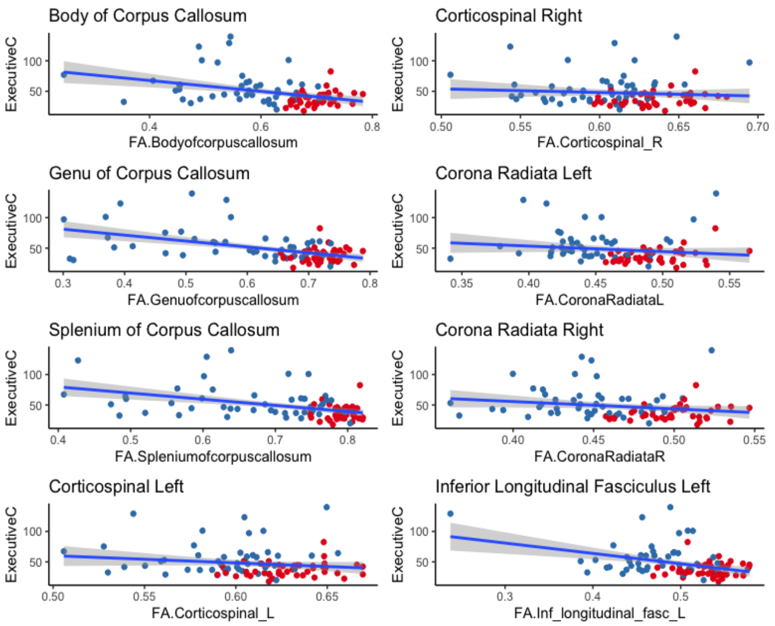

Supplementary figure 5: Correlation between mean tract FA and composite executive function (ExecutiveC) neuropsychological scores for all TBI patients (n=92). Red dots denote patients identified as having normal DTI. Blue dots denote patients identified as having abnormal DTI using the diagnostic pipeline.

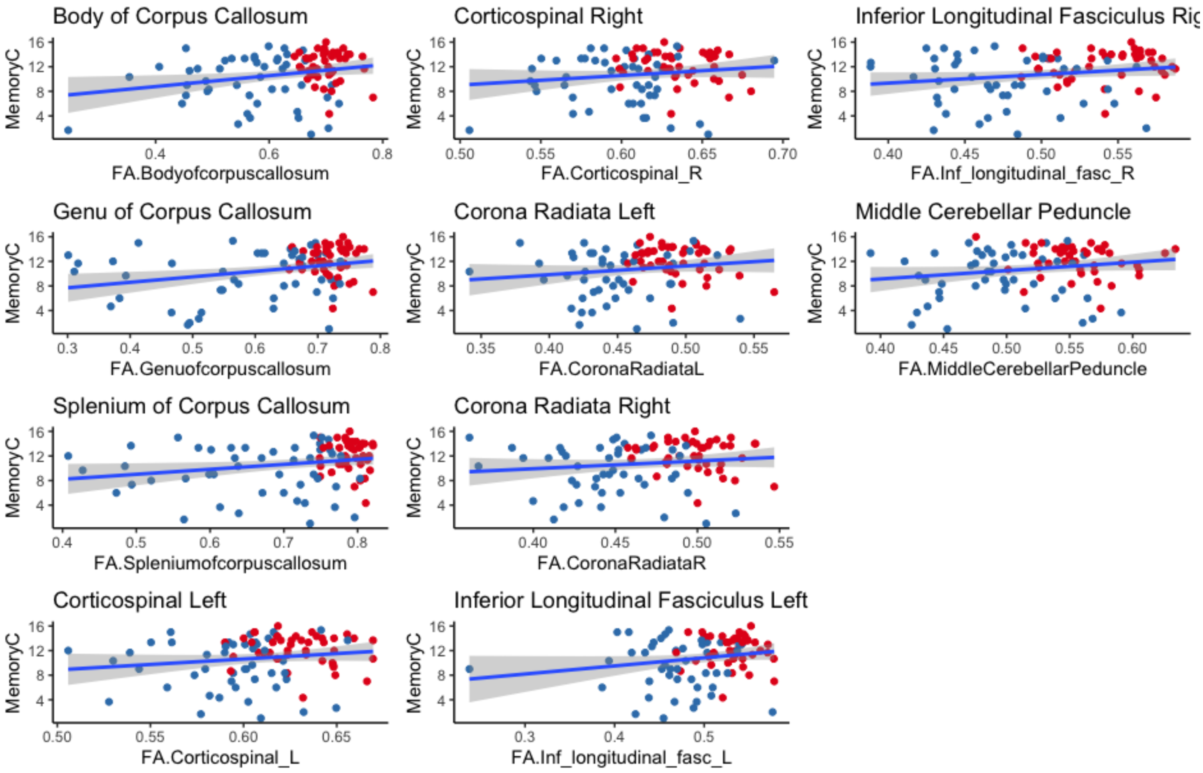

Supplementary figure 6: Correlation between mean tract FA and composite memory (MemoryC) neuropsychological scores for all TBI patients (n=92). Red dots denote patients identified as having normal DTI. Blue dots denote patients identified as having abnormal DTI using the diagnostic pipeline.

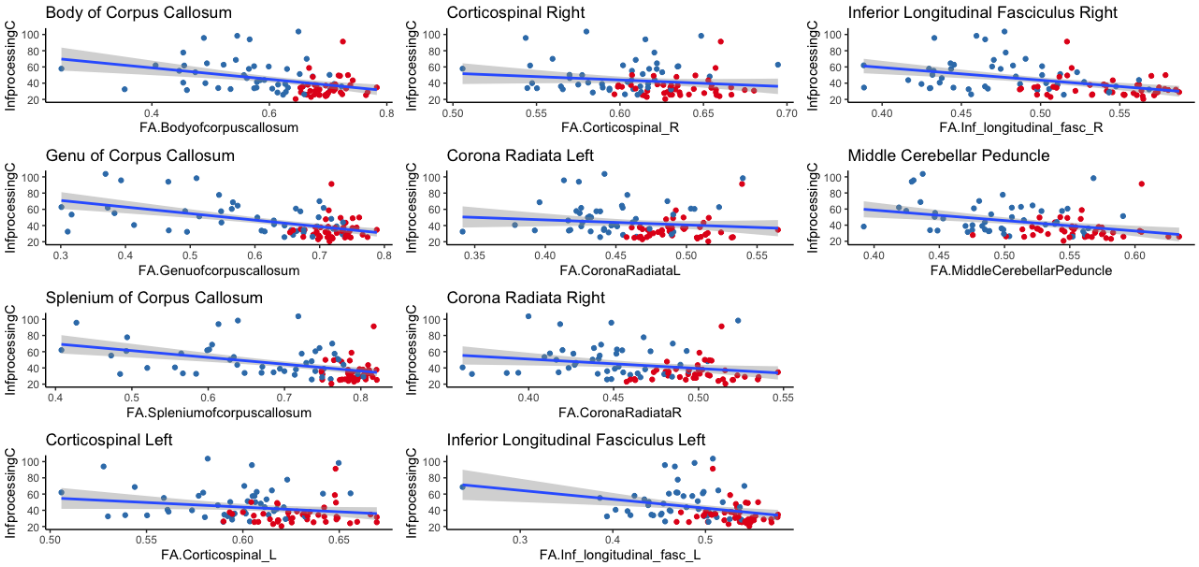

Supplementary figure 7: Correlation between mean tract FA and composite information processing (InfProcessingC) neuropsychological scores for all TBI patients (n=92). Red dots denote patients identified as having normal DTI. Blue dots denote patients identified as having abnormal DTI using the diagnostic pipeline.

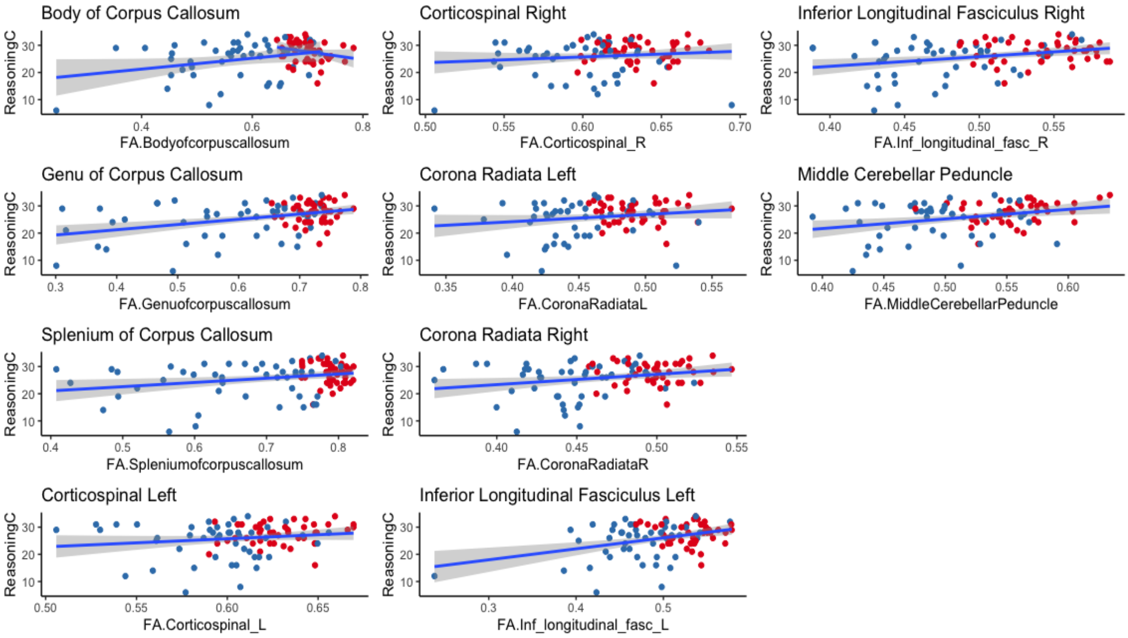

Supplementary figure 8: Correlation between mean tract FA and composite reasoning (ReasoningC) neuropsychological scores for all TBI patients (n=92). Red dots denote patients identified as having normal DTI. Blue dots denote patients identified as having abnormal DTI using the diagnostic pipeline.

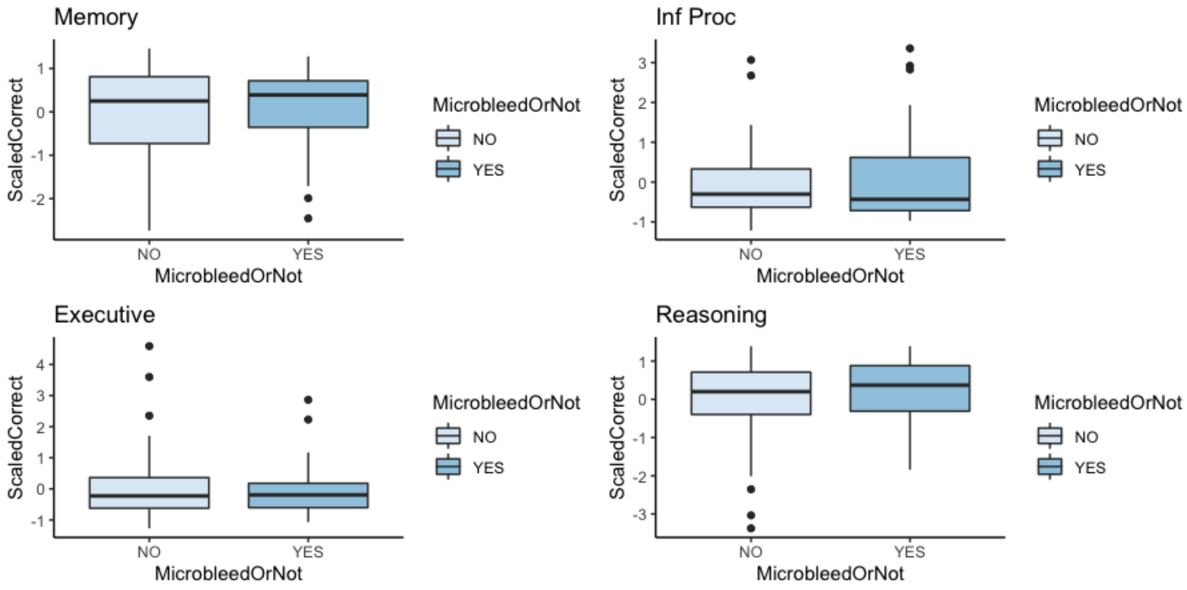

Supplementary figure 9: Comparison of cognitive performance between patients with (n=40) or without (n=52) microbleeds.

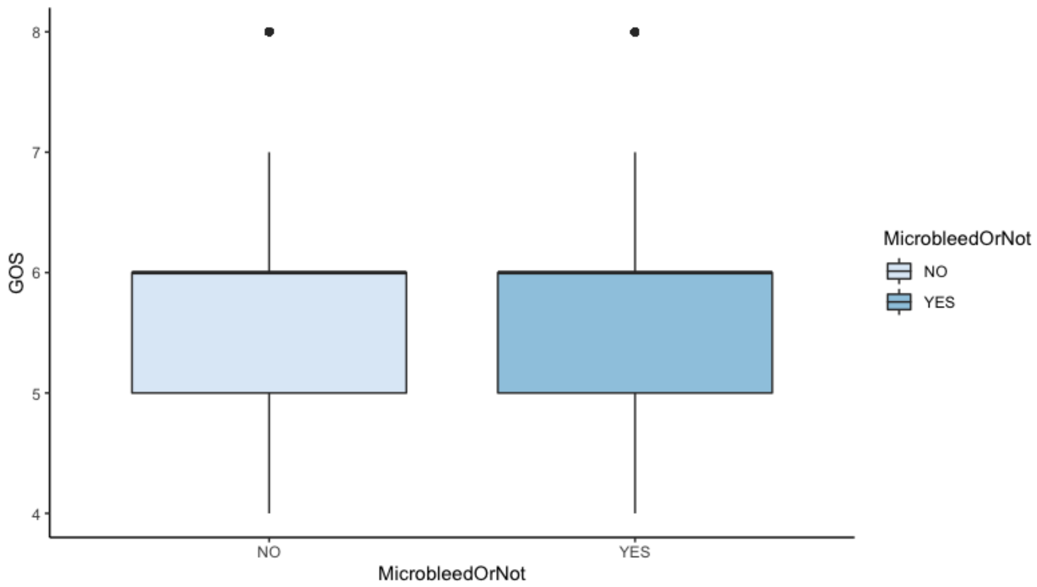

Supplementary figure 10: Comparison of functional outcome (GOSE) between patients with or without microbleeds.

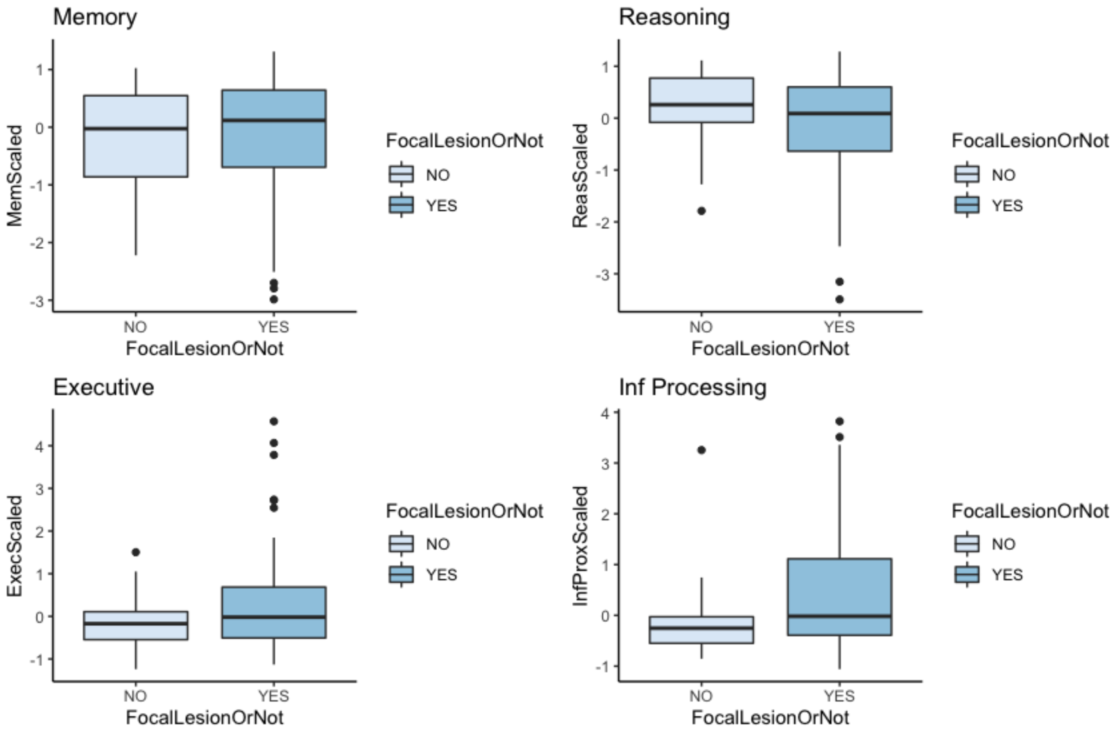

Supplementary figure 11: Comparison of cognitive performance between patients with (n=27) or without (n=65) focal lesions.

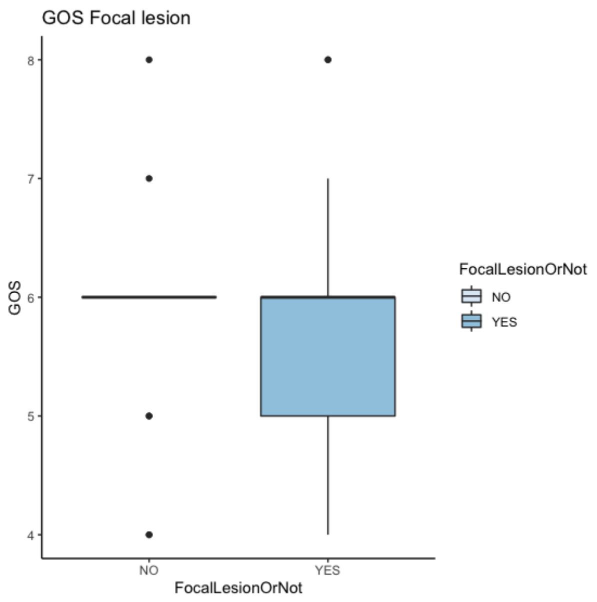

Supplementary figure 12: Comparison of functional outcomes (GOSE) between patients with or without focal lesions

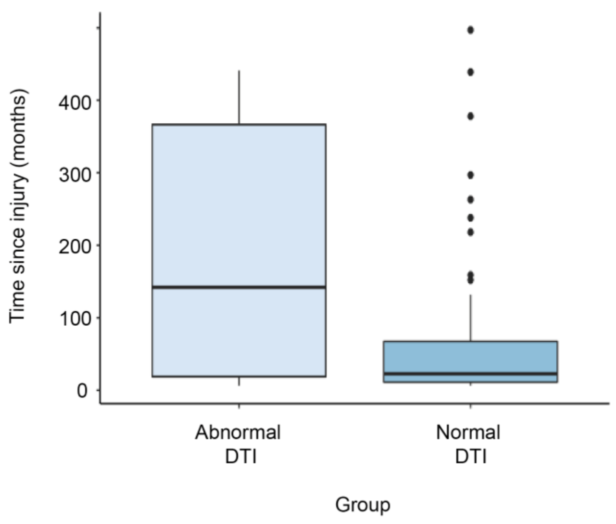

Supplementary figure 13: Comparison of time since injury (in months) between patients diagnosed with normal vs. abnormal DTI.

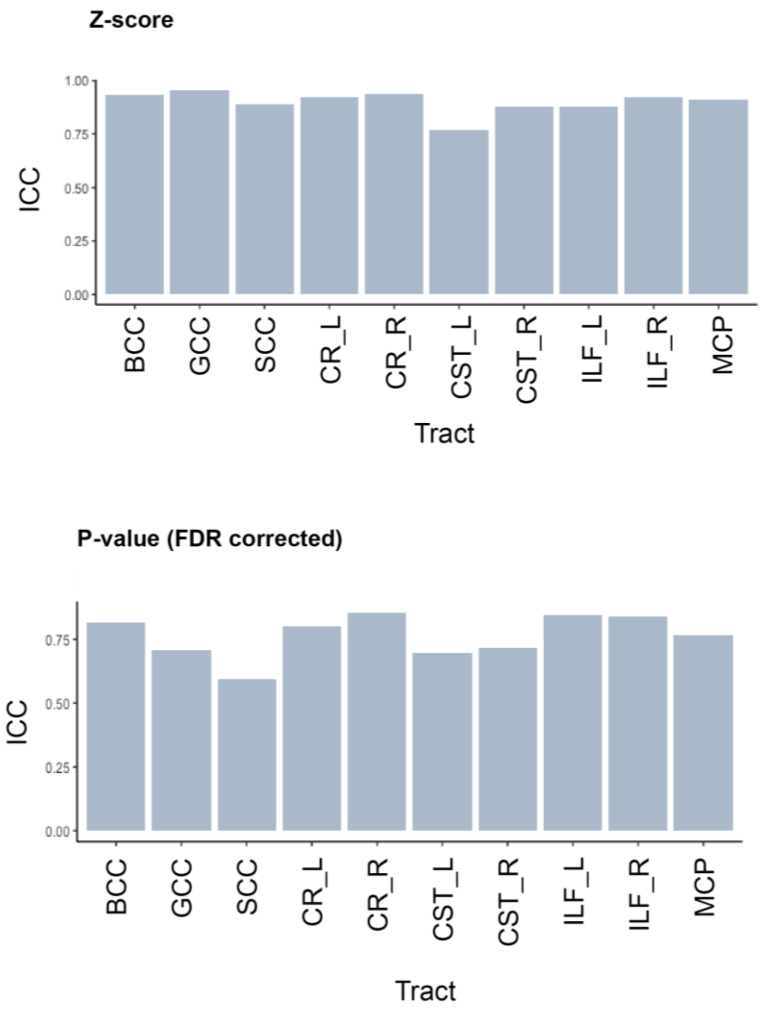

Supplementary figure 14: Intraclass correlation coefficient (ICC) of diagnostic results (z-scores and FDR corrected p values) between subacute and chronic timepoints in TBI patients.

Supplementary Tables

Age	Gender	Severity	Cause	PTA	Contusion	Microbleeds	Medication
65	M	Mod/Sev	RTA	>7 days	Y	N	nil
38	M	Mod/Sev	violence/ assault	2 days	N	Y	nil
51	F	Mod/Sev	incident fall	2 days	unknown	unknown	Lansoprazole 15mg od
31	M	Mod/Sev	RTA	90 days	Y	Y	nil
54	M	Mod/Sev	RTA	5 days	N	N	Citalopram 30mg, thyroxine 150 mcg
20	M	Mod/Sev	incident fall	28 days	N	Y	nil
36	M	Mod/Sev	violence/ assault	3 days	Y	N	nil
44	M	Mod/Sev	RTA	90 days	Y	Y	nil
51	M	Mod/Sev	RTA	several weeks	Y	Y	nil
64	M	Mod/Sev	RTA	few hours to a day	Y	N	Omeprazole 40mg, Levothyroxine 75mcg
58	M	Mod/Sev	RTA	1 month	Y	N	nil
34	M	Mod/Sev	other	6 weeks	Y	Y	nil
45	M	Mod/Sev	RTA	2 weeks	N	Y	nil
20	M	Mod/Sev	RTA	2 months	Y	Y	nil
52	M	Mod/Sev	RTA	5 days	N	Y	nil
45	M	Mod/Sev	other	14 days	N	N	nil
31	M	Mod/Sev	violence/ assault	months	Y	Y	nil
49	M	Mod/Sev	other	yes but unknown duration	Y	Y	Loperamine 2mg
24	M	Mod/Sev	RTA	14 days	N	N	nil
52	M	Mod/Sev	incident fall	21 days	Y	Y	Levetirecetam 1250mg bd, perindopril 2mg od
54	M	Mod/Sev	violence/ assault	15 days	Y	Y	nil
26	M	Mod/Sev	violence/ assault	56 days	Y	N	Amitriptyline 10mg, Sodium Valproate 100mg
21	F	Mod/Sev	RTA	35 days	N	Y	Fluoxetine 20mg
39	F	Mod/Sev	incident fall	2 days	N	N	nil
47	M	Mod/Sev	RTA	3 days	Y	Y	nil
22	M	Mod/Sev	RTA	450 days	Y	Y	nil
34	M	Mod/Sev	incident fall	0 days	Y	N	nil
48	M	Mod/Sev	RTA	60 days	Y	N	Amitriptyline 20mg tds, Fexofenadine 180mg od,<br>Esomeprazole 40mg od

							Solifenacin 5mg od<br>Loperamine 10mg od
38	M	Mod/Sev	violence/ assault	700 days	Y	N	nil
49	F	Mod/Sev	violence/ assault	10 days	Y	N	Levothyroxne 150mcg od, omeprazole
36	F	Mod/Sev	RTA	120 days	Y	Y	nil
43	M	Mod/Sev	RTA	42 days	N	Y	nil
31	M	Mod/Sev	violence/ assault	21 days	Y	N	Growth Hormone 2mg, Lamotrigine 50mg bd
37	M	Mod/Sev	incident fall	120 days	Y	N	Tegretol CR 400mg BD
33	M	Mod/Sev	RTA	30 days	Y	Y	Citalopram 10mg OD, Baclofen 20mg OD
35	M	Mod/Sev	incident fall	7 days	Y	Y	nil
33	M	Mod/Sev	RTA	28 days	Y	N	Symbicort
52	M	Mod/Sev	violence/ assault	4 days	Y	N	Lansoprazole 15mg od
38	M	Mod/Sev	violence/ assault	14 days	unknown	unknown	nil
31	M	Mod/Sev	RTA	120 days	unknown	unknown	nil
39	M	Mod/Sev	RTA	2 weeks	Y	N	nil
24	F	Mod/Sev	RTA	120 days	unknown	unknown	Citalopram 40mg OD
35	M	Mod/Sev	incident fall	3 days	unknown	unknown	Salbutamol, Beclomethasone
42	M	Mod/Sev	RTA	unknown	N	N	Modafinil 300od, citalopram 20 OD,
37	M	Mod/Sev	RTA	4 days	Y	N	Omeptrazol 40mg OD, Gabapentin 900mg OD
6	M	Mod/Sev	incident fall	4-6 hours	N	N	nil
59	M	Mod/Sev	RTA	7 days	N	Y	nil
57	F	Mod/Sev	incident fall	1-7 days	Y	Y	Demeclocycline 300g bd, ramipril 5mg od,<br>salbutamol brn,
54	M	Mod/Sev	incident fall	2 days	Y	N	nil
39	M	Mod/Sev	RTA	unknown	N	Y	nil
44	M	Mod/Sev	RTA	6 days	Y	Y	unknown
48	M	Mod/Sev	violence/ assault	24-48 hours	N	N	Carbamezipine 200mg
65	M	Mod/Sev	unknown	unknown	Y	Y	unknown
59	M	Mod/Sev	RTA	1-7 days	Y	N	Lansoprazole 30mg od
37	M	Mod/Sev	incident fall	1 days	Y	N	nil
26	M	Mod/Sev	RTA	3 days	Y	Y	nil
24	M	Mod/Sev	RTA	unknown	Y	N	nil

39	M	Mod/Sev	violence/ assault	3 weeks	Y	N	nil
53	M	Mod/Sev	incident fall	unknown	Y	Y	nil
29	F	Mod/Sev	RTA	still in PTA	Y	Y	Lansoprasine 30mg, Phenytoin 300mg,
51	M	Mod/Sev	unknown	unknown	Y	Y	Gabapentin 300mg tds, Amitriptyline 50 mg
52	M	Mod/Sev	Incident fall	24-48 hours	Y	Y	nil
57	M	Mod/Sev	RTA	4 days	Y	N	unknown
40	M	Mod/Sev	RTA	42 days	Y	N	nil
42	F	Mod/Sev	RTA	3 days	Y	Y	nil
40	F	Mod/Sev	unknown	unknown	Y	N	unknown
31	M	Mod/Sev	incident fall	unknown	N	Y	nil
34	M	Mod/Sev	unknown	3 days	Y	N	nil
49	F	Mod/Sev	incident fall	24-48 hours	Y	N	Ibuprofen
39	M	Mod/Sev	unknown	7 days	Y	N	ramipril
44	F	Mod/Sev	RTA	unknown	Y	N	unknown
49	M	Mod/Sev	RTA	unknown	unknown	unknown	nil
61	F	Mod/Sev	RTA	120 days	Y	Y	Citalopram 10mg od
39	F	Mod/Sev	RTA	unknown	Y	Y	citalopram 20mg OD
49	M	Mod/Sev	unknown	unknown	Y	N	phenytoin 35omg OD, Omnitrope GH 0.3mg/0.4mg, Bendroflumethiazide 2.5mg, Thiamine 300mg OD, Quetiapine 100mg OD, Mirtazapine 45mg OD, Atorvastatin 40mg OD, Amlodipine 10mg OD, Ramipril 7.5mg OD, Pregabalin 300mg OD
72	M	Mod/Sev	unknown	unknown	Y	N	nil
40	F	Mod/Sev	RTA	unknown	Y	N	Tegretol, propranolol
65	M	Mod/Sev	incident fall	14 days	N	N	amlodipine, simvastatin, ramipril,
54	M	Mod/Sev	RTA	10 days	Y	Y	Tegretol 200mg BD, Amytriptyline 100mg
56	F	Mod/Sev	RTA	several months	Y	N	ventolin, seretide
43	M	Mod/Sev	RTA	unknown	Y	N	pregabalin 75mg bd, ventolin, cetirizine, omeprazole, lamotrigine 50mg bd
39	M	Mod/Sev	RTA	unknown	Y	N	tegretol 400mg bd
57	M	Mod/Sev	RTA	unknown	Y	N	nil
31	F	Mod/Sev	unknown	unknown	unknown	unknown	unknown

38	M	Mod/Sev	unknown	unknown	unknown	unknown	unknown
51	M	Mod/Sev	incident fall	unknown	unknown	unknown	nil
54	M	Mod/Sev	RTA	1 days	N	N	nil
46	F	Mod/Sev	incident fall	4 weeks	unknown	unknown	nil
43	M	Mod/Sev	RTA	no	N	N	nil
55	M	Mod/Sev	violence/ assault	4 days	N	N	Levetiracetam, Salbutamol
48	M	Mod/Sev	RTA	no	N	N	Salbutamol, Seretide
57	M	Mod/Sev	RTA	no	N	N	nil

Supplementary table 1: Patient demographics. M=male, F=female. RTA=road traffic accident, PTA=post traumatic amnesia. Bd= twice a day, OD=once daily.

Subj	Age	Gender	Severity	Mechanism of injury	PTA duration	Time since injury at acute (days)	Time since injury at sub-acute (days)
1	22	Male	Mod-severe	RTA	1to7days	11	170
2	24	Male	Mod-severe	Assault	NK	26	179
3	45	Male	Mod-severe	RTA	1to7days	29	191
4	41	Male	Mod-severe	Assault	None	28	188
5	55	Female	Mod-severe	Fall	1to24Hour	29	249
6	56	Female	Mod-severe	RTA	NK	49	175
7	40	Male	Mod-severe	Assault	1to24Hour	50	189
8	52	Male	Mod-severe	RTA	1to29Minute	29	191
9	70	Male	Mod-severe	Fall	1to24Hour	25	193
10	58	Male	Mod-severe	Fall	1to24Hour	13	196
11	42	Male	Mod-severe	Fall	None	28	226
12	67	Female	Mod-severe	RTA	1to7days	20	183
13	26	Male	Mod-severe	Fall	None	13	181
14	60	Female	Mod-severe	Fall	None	12	186
15	43	Male	Mod-severe	Fall	1to24Hour	25	203
16	47	Male	Mod-severe	Fall	None	22	183
17	45	Male	Mod-severe	RTA	1to24Hour	16	190
18	52	Female	Mod-severe	RTA	1to7days	44	177
19	49	Male	Mod-severe	Fall	1to7days	26	219
20	75	Female	Mod-severe	Fall	None	44	183
21	70	Male	Mod-severe	RTA	1to7days	14	196
22	44	Male	Mod-severe	Fall	NK	24	166
23	47	Male	Mod-severe	Fall	1to7days	21	176
24	41	Female	Mod-severe	Fall	NK	20	235
25	60	Male	Mod-severe	Sports	NK	35	203

Supplementary table 2: Patient demographics of additional longitudinal acute TBI patient cohort (n=25). M=male, F=female. RTA=road traffic accident, PTA=post traumatic amnesia. NK=not known.

Tract	Abbreviation
Anterior limb of internal capsule L	ALIC_L
Anterior limb of internal capsule R	ALIC_R
Body of corpus callosum	CCB
Cerebral peduncle L	CP_L
Cerebral peduncle R	CP_R
Cingulate_cing_L	CC_L
Cingulate_cing_R	CC_R
Cingulum_Hippocampus_L	CH_L
Cingulum_Hippocampus_R	CH_R
Corona Radiata L	CR_L
Corona Radiata R	CR_R
Corticospinal_L	CST_L
Corticospinal_R	CST_R
External capsule L	EXC_L
External capsule R	EXC_R
Fornix	Fnx
Fornix cres L	FC_L
Fornix cres R	FC_R
Genu of corpus callosum	CCG
Inf_fronto-occipital_fasc_L	IFOF_L
Inf_fronto-occipital_fasc_R	IFOF_R
Inf_longitudinal_fasc_L	ILF_L
Inf_longitudinal_fasc_R	ILF_R
Inferior cerebellar peduncle L	ICP_L
Inferior cerebellar peduncle R	ICP_R
Medial lemniscus L	ML_L
Medial lemniscus R	ML_R
Middle Cerebellar Peduncle	MCP
Pontine crossing tract	PCT
Posterior limb of internal capsule L	PLIC_L
Posterior limb of internal capsule R	PLIC_R
Posterior thalamic radiation L	PTR_L
Posterior thalamic radiation R	PTR_R
Retrolenticular part of internal capsule L	RLIC_L
Retrolenticular part of internal capsule R	RLIC_R
Splenium of corpus callosum	CCS
Superior cerebellar peduncle L	SCP_L
Superior cerebellar peduncle R	SCP_R

Superior fronto-occipital fasciculus L	SFOF_L
Superior fronto-occipital fasciculus R	SFOF_R
Superior longitudinal fasciculus L	SLF_L
Superior longitudinal fasciculus R	SLF_R
Tapetum L	TP_L
Tapetum R	TP_R
Uncinate fasciculus L	UF_L
Uncinate fasciculus R	UC_R

Supplementary table 3: List of candidate tracts and associated acronyms from the ICBM-DTI-81 white matter atlas (Oishi et al., 2008).

Patient	No. Abnormal tracts including lesion	No. Abnormal tracts excluding lesion
1	6	6
2	3	3
3	0	0
4	0	0
5	0	0
6	9	9
7	1	1
8	5	5
9	5	5
10	9	8
11	7	5
12	9	9
13	0	0
14	3	0
15	0	0
16	7	6
17	0	0
18	8	8
19	6	6
20	1	1
21	0	0
22	5	4
23	6	6
24	0	0
25	0	0
26	0	0
27	0	0
28	4	3
29	0	0
30	9	9
31	0	0
32	0	0
33	6	6
34	0	0
35	5	5
36	5	3
37	0	0
38	8	6
39	0	0
40	2	0
41	0	0

42	0	0
43	0	0
44	5	4
45	0	0
46	0	0
47	6	5
48	9	9
49	9	8
50	9	9
51	5	3
52	4	4
53	9	9
54	9	8
55	10	10
56	0	0
57	1	1
58	1	1
59	3	2
60	1	0
61	0	0
62	0	0
63	0	0
64	6	5

Supplementary table 4: Diagnostic results of the 64 patients with focal lesions when including or excluding lesions within the DTI diagnostic pipeline.

Patient	Routine MRI findings	No. of abnormal tracts	No. abnormal tracts (accounting for age)	Whole brain white matter skeleton	Whole brain white matter skeleton (accounting for age)
1	Contusion	6	7	Abnormal	Abnormal
2	Microbleed	0	0	Normal	Normal
3	Contusion & Microbleed	3	7	Abnormal	Abnormal
4	No visible damage	3	3	Normal	Normal
5	Microbleed	9	9	Abnormal	Abnormal
6	Contusion	0	0	Normal	Normal
7	Contusion & Microbleed	0	0	Normal	Normal
8	Contusion & Microbleed	0	0	Normal	Normal
9	Contusion	9	9	Abnormal	Abnormal
10	Contusion & Microbleed	1	4	Normal	Abnormal
11	Microbleed	0	0	Normal	Normal
12	Contusion & Microbleed	5	5	Abnormal	Abnormal
13	Microbleed	0	0	Normal	Normal
14	No visible damage	0	0	Normal	Normal
15	Contusion & Microbleed	5	7	Abnormal	Abnormal
16	Contusion & Microbleed	9	9	Abnormal	Abnormal
17	No visible damage	0	0	Normal	Normal
18	No visible damage	0	0	Normal	Normal
19	Contusion	7	7	Abnormal	Abnormal
20	Contusion & Microbleed	9	10	Abnormal	Abnormal
21	Contusion & Microbleed	0	0	Normal	Normal
22	Contusion	3	5	Abnormal	Abnormal
23	Microbleed	5	7	Abnormal	Abnormal
24	Contusion & Microbleed	0	0	Normal	Normal
25	Contusion & Microbleed	7	9	Abnormal	Abnormal
26	Contusion	0	0	Normal	Normal
27	Contusion	8	9	Abnormal	Abnormal
28	Contusion	6	6	Normal	Normal
29	No visible damage	0	0	Normal	Normal
30	Contusion & Microbleed	1	1	Normal	Normal
31	Microbleed	3	3	Normal	Normal

32	Contusion	0	0	Normal	Normal
33	Contusion	5	5	Abnormal	Abnormal
34	Contusion & Microbleed	6	6	Normal	Normal
35	Contusion & Microbleed	0	0	Normal	Normal
36	Contusion	0	0	Normal	Normal
37	Contusion	0	0	Normal	Normal
38	Contusion & Microbleed	0	0	Normal	Normal
39	Contusion & Microbleed	4	7	Abnormal	Abnormal
40	Contusion	0	0	Normal	Normal
41	Contusion & Microbleed	9	9	Abnormal	Abnormal
42	Contusion	0	0	Normal	Normal
43	No visible damage	0	0	Abnormal	Abnormal
44	No visible damage	0	0	Normal	Normal
45	Contusion	0	0	Normal	Normal
46	No visible damage	1	1	Normal	Normal
47	Microbleed	4	4	Normal	Normal
48	Contusion & Microbleed	6	6	Abnormal	Normal
49	Contusion	0	0	Normal	Normal
50	Microbleed	0	0	Normal	Normal
51	Contusion & Microbleed	5	6	Normal	Abnormal
52	No visible damage	0	0	Normal	Normal
53	Contusion & Microbleed	5	3	Abnormal	Normal
54	Contusion	0	0	Normal	Normal
55	Contusion	8	7	Abnormal	Abnormal
56	Contusion & Microbleed	0	0	Normal	Normal
57	Contusion	2	2	Normal	Normal
58	Contusion	0	0	Normal	Normal
59	Contusion & Microbleed	0	0	Normal	Normal
60	Contusion & Microbleed	0	0	Normal	Normal
61	Contusion & Microbleed	5	5	Abnormal	Abnormal
62	Contusion	0	0	Normal	Normal
63	Contusion	0	0	Normal	Normal
64	Contusion	6	6	Abnormal	Abnormal
65	No visible damage	0	0	Normal	Normal
66	Microbleed	0	0	Normal	Normal

67	No visible damage	0	0	Normal	Normal
68	Contusion	9	9	Abnormal	Abnormal
69	Microbleed	3	3	Abnormal	Abnormal
70	Contusion & Microbleed	9	8	Abnormal	Abnormal
71	Contusion & Microbleed	9	9	Abnormal	Abnormal
72	Contusion	5	6	Abnormal	Abnormal
73	Contusion	4	3	Abnormal	Abnormal
74	Contusion	9	9	Abnormal	Abnormal
75	No visible damage	10	10	Abnormal	Abnormal
76	Contusion & Microbleed	9	9	Abnormal	Abnormal
77	Contusion	10	10	Abnormal	Abnormal
78	Contusion	0	0	Normal	Normal
79	Contusion	1	1	Normal	Normal
80	Contusion & Microbleed	1	1	Normal	Normal
81	Contusion	3	3	Abnormal	Abnormal
82	No visible damage	5	5	Normal	Normal
83	No visible damage	0	0	Normal	Normal
84	No visible damage	9	9	Abnormal	Abnormal
85	No visible damage	0	0	Normal	Normal
86	Contusion	1	1	Normal	Normal
87	Contusion & Microbleed	0	0	Normal	Normal
88	Contusion	0	0	Normal	Normal
89	Microbleed	0	0	Normal	Normal
90	Contusion	0	0	Normal	Normal
91	Contusion	0	0	Normal	Normal
92	Contusion	6	6	Abnormal	Abnormal

Supplementary table 5: Individual patient diagnostic results with and without accounting for age using the DTI diagnostic pipeline.

**Neuropsychological tests results by group**				
**Cognitive domain**	**Cognitive variable**	**TBI (mean ± SD) (n=92)**	**Control (mean ± SD) (n=33)**	**P Value (FDR corrected)**
Abstract reasoning	WASI matrix reasoning	25.82 ± 5.87	27.42 ± 6.01	p=0.116
Associative memory	People test total	22.28 ± 8.07	27.24 ± 6.52	p=0.002**
	People test delayed	7.96 ± 3.47	9.81 ± 2.92	p=0.008**
	People test forgetting	2.07 ± 2.09	1.51 ± 2.43	p=0.060
Processing speed	Trail Making Test Trail A (s)	33.58 ± 18.55	21.11 ± 9.02	P<0.001***
	Trail Making Test Trails B (s)	76.36 ± 47.32	47.70 ± 22.88	P<0.001***
	Colour naming (s)	35.05 ± 7.94	27.97 ± 5.15	P<0.001***
	Word reading (s)	25.54 ± 6.30	20.52 ± 4.65	P<0.001***
Executive Function	Trail Making Test Trails B minus A (s)	42.77 ± 34.91	26.58 ± 18.54	p=0.009**
	Inhibition/switching (s)	74.75 ± 29.35	58.07 ± 16.08	p<0.001***
	Inhibition/switching minus a baseline of colour naming and word reading	37.35 ± 32.15	30.69 ± 18.18	p=0.384

Supplementary table 6: Comparison of cognitive performance between TBI patients (n=92) and healthy controls (n=35). *=p<0.05, **=p<0.01,***=p<0.001.
